# Genetic analyses reveal independent domestication origins of the emerging oil crop *Paeonia ostii*, a tree peony with a long-term cultivation history

**DOI:** 10.1038/s41598-017-04744-z

**Published:** 2017-07-13

**Authors:** Li-Ping Peng, Chang-Fu Cai, Yuan Zhong, Xing-Xing Xu, Hong-Li Xian, Fang-Yun Cheng, Jian-Feng Mao

**Affiliations:** 1 National Flower Engineering Research Centre, College of Landscape Architecture, National Engineering Laboratory for Tree Breeding, Key Laboratory of Genetics and Breeding in Forest Trees and Ornamental Plants, Ministry of Education, College of Biological Sciences and Technology, Beijing Forestry University, Beijing, China; 2 Forestry Department of Shaanxi Province, Xian, China

## Abstract

*Paeonia ostii*, a member of tree peony, is an emerging oil crop with important medical and oil uses and widely cultivated in China. Dissolving the genetic diversity and domestication history of this species is important for further genetic improvements and deployments. We firstly selected 29 simple sequence repeats (SSRs) via transcriptome mining, segregation analyses and polymorphism characterizations; then, 901 individuals from the range-wide samples were genotyped using well-characterized SSR markers. We observed moderate genetic diversity among individuals, and Shaanxi Province was identified as the center of genetic diversity for our cultivated plants. Five well-separated gene pools were detected by STRUCTURE analyses, and the results suggested that multiple independent domestication origins occurred in Shaanxi Province and Tongling City (Anhui Province). Taken together, the genetic evidence and the historical records suggest multiple long-distance introductions after the plant was domesticated in Shandong, Henan and Hunan provinces. The present study provides the first genetic evaluation of the domestication history of *P. ostii*, and our results provide an important reference for further genetic improvements and deployments of this important crop.

## Introduction

Edible oils represent an important food source for the rapidly growing global population^1, 2^. With the increasing demand for fats and oils, researchers have targeted plant sources to explore their uses and functional properties^3^ with the goal of developing improved varieties of edible oils that can withstand future challenges, such as climate change, sustainability and food security^4^. *Paeonia ostii* is a traditional medicinal plant that represents a newly discovered and underutilized woody oilseed plant with a considerable agronomic potential for edible oil production. Originally, this species was grown for the root bark, which is used as an antispasmodic throughout Asia^5^. Recent studies have indicated that this species presents a competitive agronomic performance as compared to established oil crops for its high seed yield and unsaturated fatty acids content especially high *α*-linolenic acid content in the seed oil^6, 7^. Grain yields of over 13.34 kg/ha have been achieved with common cultivars of *P. ostii*, and a considerable variation in oil content in seed has been recorded in this species, with values ranging from 20–35%^8, 9^. *α*-Linolenic acid, which is a necessary element that cannot be produced within the human body and must be acquired through diet, is commonly found at unusually high levels (above 40%)^10–12^ in the seed oil of *P. ostii*, which indicates that this plant is a valuable crop for linolenic acid production^13^. Moreover, seed oil of *P. ostii* is a versatile oil as its use is not limited to food, but it is also widely used in non-food applications, such as in oleochemicals, cosmetics, and pharmaceuticals^14, 15^. Therefore, *P. ostii* is an important economic plant with simultaneous medicinal and oil applications. The rediscovery of the value of this oil crop and its rapid deployment in recent years represent unprecedented opportunities for farmers and have attracted considerable interest among plant breeders^10, 16, 17^.

*P. ostii* belongs to the section *Moutan* of the genus *Paeonia* and the family Paeoniaceae^18^, and this diploid tree peony species, with 2n = 2x = 10 chromosomes, is indigenous to China^19^. This plant is cross-pollinated by insects and propagated by seeds^20^. Compared with other tree peonies in this section, *P. ostii* is clearly characterized with white or pale rose petals without the base blotch, and the leaves contain no more than fifteen leaflets and present red or purple filaments, a disc and a stigma^21^. Literature on its cultivation has been recorded since the Qin to Han dynasties (>2,000 years ago), and its root bark was originally used as a medicine. However, the plant was not used for gardening^22^, although it may represent one of the parental species for breeding the popular ornamental tree peony^23, 24^. Currently, *P. ostii* is being cultivated at high concentrations in Bozhou and Tongling city in Anhui Province and the southwest region of Hunan Province, and these areas supply thousands of kilograms of root bark materials for the medical industry annually. In addition, this species is sparsely cultivated across southern and northern China, mainly in Shanxi, Shandong and Shaanxi provinces. Shaanxi Province is often reported as the region with the largest species diversity of *Paeonia* ^25^, and in this region, the largest number of wild populations of different woody and herb peonies have been reported^26^. In addition, historical literature records Shaanxi as the original cultivation location of *P. ostii* ^27, 28^. However, the cultivation history of *P. ostii* remains largely unclear. Studies have indicated that *P. ostii* cultivation originated in the Fenghuang Mountain area of Tongling city in Anhui Province^29, 30^. However, multiple independent cultivations may have also occurred in Henan Province, which has represented a famous location for the cultivation and breeding of the ornamental peony for more than 1,000 years^5, 31^. *P. ostii* is an important rootstock resource for grafting the ornamental tree peony and one of the parents for the ornamental tree peony^30^. The current specimens cited in the original taxonomic description were derived from plants collected in three Chinese provinces: Henan, Hunan and Shaanxi^28^. Recent searches for the species as a wild plant have been largely unsuccessful^32, 33^. The plant may have been common in the wild at one time but has suffered from over-collection of its roots for use as a Chinese medicine^32^. When phenotypic variation was qualified across the popular cultivars^34^, little information was obtained on the divergence and relationship among cultivars. However, genetic evaluations may help to clarify the genetic diversity and relationship among the populations and provide insights on the domestication history and future breeding.

In recent years, *P. ostii* has been cultivated as a new crop for edible oil production, which represents an unprecedented opportunity for the peony oil industry; however, the genetic background of this species is poorly understood. Thus, proper initiation of breeding populations by the selection of optimal genotypes from the available germplasm is lacking and a proper resource management strategy across the entire country has not been developed. These questions present the main challenges for the deployment of *P. ostii*. Hence, comprehensive information on the genetic diversity and population structure of *P. ostii* is urgently required.

Population genetics analyses rely primarily on microsatellite markers, which are also powerful tools for marker-assisted selection (MAS) in plant breeding. Co-dominant inheritance, a high level of transferability, close associations with genes of known function and a low cost of development are some of the advantages that led to an increased focus on simple sequence repeat (SSR) markers^35, 36^. To date, more than 500 SSR markers have been developed for the *Moutan* section^37–46^. Segregation analyses that incorporate genetic mapping may provide a good strategy for selecting a set of optimal SSR markers. These markers can then be used to determine the allele inheritance and independent assortment of *P. ostii* ^47^ and for conducting a population genetic study.

Although the population structure and genetic diversity of *P. ostii* are still unknown, a complex pattern of population divergence is expected because of the long domestication history and outcrossing property of these plants. *P. ostii* presents an opportunity to investigate the genetic effects of migration, hybridization, adaptive divergence and anthropogenic influence on the genetic diversification of outcrossing woody plants. Clarifying the genetic variation could provide valuable information for the proper management of *P. ostii* genetic resources as well as for the deployment of these resources and directive breeding. We first screened a set of SSR markers that are suitable for population genetic evaluations based on segregation analyses, amplifications and polymorphic properties. Then, we performed a population genetic evaluation of a large collection of samples collected over a wide range in China. The goal of our research was to understand the genetic diversity among and within the population and the genetic structure among populations, infer the domestication history, and provide information for genetic assessments, regional genetic improvements and management of this emerging oil crop.

## Results

### SSR markers, segregation and genetic map construction

**Figure 1:**
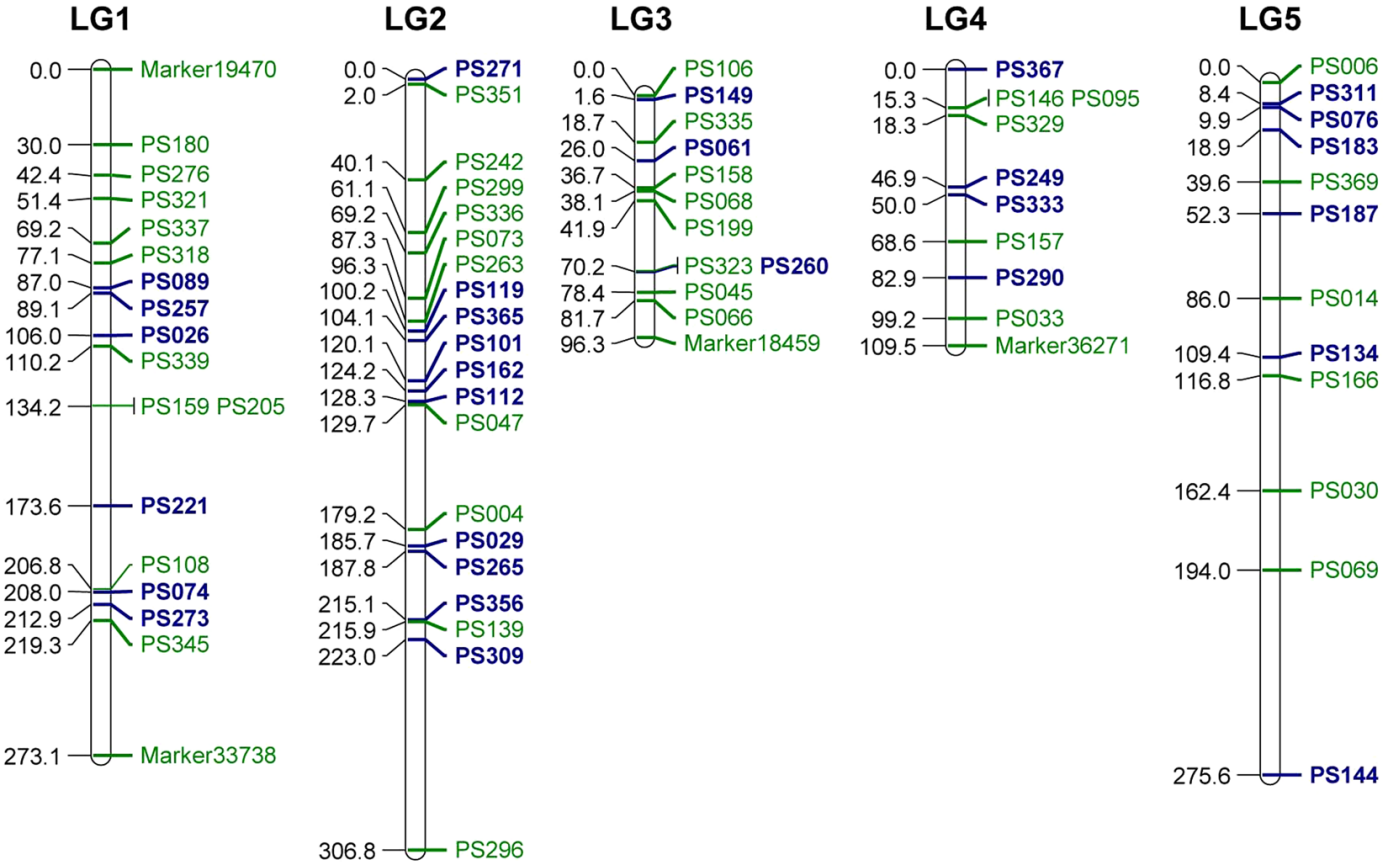
EST-SSR based genetic linkage map of tree peony. Blue indicate the 29 polymorphic markers of *P. ostii* used in this study. The names of the linkage groups are labeled on top, the genetic distances of the loci (cM) are shown on the right, and the names of loci are shown on the left of the linkage groups.

The transcriptome assembly of the tree peony (*Paeonia suffruticosa* ‘LuoYang Hong’)^48^, which is a related species related to *P. ostii*, was mined for SSR markers. In total, 2,989 SSR loci were detected, and 788 SSR loci were selected and tested. Of these loci, 373 yielded fragments of expected sizes^43^. In addition, we developed a F_1_ mapping population for segregation analyses and genetic map construction. This mapping population included 195 progenies derived from a cross between *P. ostii* ‘FenDanBai’ (female parent) and *P. × suffruticosa* ‘HongQiao’ (male parent). We then tested the 373 validated SSR loci in our mapping population and determined that 74 SSRs were polymorphic loci (Table S2). The chi-squared (χ^2^) segregation test was performed and the genetic map was constructed using Joinmap 4.1^49^. In total, 68 SSR loci were sorted into 5 segregation types (9 SSRs, ef × eg type; 3 SSRs, hk × hk type; 8 SSRs, lm × ll type; 47 SSRs, nn × np type; and 1 SSR, ab × cd type) and successfully mapped to 5 linkage groups. Among these 68 SSR loci, 48 present Mendelian inheritance (Fig. 1, Table S2).

### Selection of microsatellites for population genetic analyses

Of the 68 mapped SSR loci, 40 showed polymorphisms among 902 sampled individuals of generally cultivated populations of *P. ostii* (Fig. 1, Table S2). Using Micro-Checker v2.2.3^50^, we detected the presence of null alleles for 7 (PS276, PS068, PS205, PS106, PS318, PS199 and PS006) of the 40 SSR loci. Furthermore, Ewen-Watterson tests performed using Arlequin v3.5.2.2^51^ targeted 4 loci (PS0337, PS158, PS004 and PS108) that deviated significantly from a neutral equilibrium model. Additionally, an analysis using Genecap v1.4^52^ determined that a pair of samples had an identical genotype; therefore, one member of this pair was removed. Finally, 29 markers (19 in Mendelian inheritance) considered unlinked and neutral with all 901 samples were remained for further analyses.

### Genetic diversity and differentiation

A total of 142 alleles were found, and the number of alleles per locus (*N*) ranged from 2 to 8, with an average of 4.897 alleles per locus. The mean polymorphism level of the loci (*PIC*) was 0.318, with a range from 0.108 (at the locus PS183) to 0.613 (PS112) (Table S3). The observed heterozygosity (*H*_*O*_) ranged from 0.080 (at the locus PS074) to 0.801 (PS101), with an average of 0.343, and the expected heterozygosity (*H*_*E*_) ranged from 0.081 (at the locus PS183) to 0.625 (PS112), with an average of 0.321 (Table S3). At the population level, 39 private alleles (*P*_*A*_) were found at 20 loci distributed in the 14 populations (Table S4). The average genetic diversity (*H*) and allelic richness (*A*_*R*_) were 0.330 and 2.194, with *H* ranging from 0.286 for *SXSLA* to 0.415 for *SXFX* (two populations from Shaanxi Province) and *A*_*R*_ ranging from 1.610 for *XXFH* (an isolated population in the south) to 2.510 for *SXSL* (a population from Shaanxi Province). We observed that the genetic diversity and allelic richness were higher in populations from Shaanxi Province (Table 1). The fixation inbreeding coefficients (*F*_*IS*_) at the locus and population levels were −0.053 and −0.045 (Table 1 and S3). The negative average fixation index (*F*) values indicated an excess of heterozygotes in *P. ostii*, which is consistent with the self-incompatibility system of this species.

**Table 1:** Population level genetic diversity estimates based on 29 SSR loci.

Population	*N*	*N*_*a*_	*N*_*e*_	*I*	*H*_*O*_	*H*_*E*_	*F*	*PPL*	*F*_*IS*_	*PIC*	*H*	*A*_*R*_	*P*_HWE_
*AHTLA*	27	2.138	1.556	0.498	0.339	0.317	−0.042	96.55%	−0.049	0.263	0.319	2.140	0.262
*AHTLB*	30	2.207	1.603	0.533	0.354	0.338	−0.004	96.55%	−0.037	0.292	0.353	2.180	0.757
*AHTLC*	29	2.276	1.594	0.508	0.340	0.320	−0.052	96.55%	−0.042	0.275	0.335	2.200	0.665
*AHTLD*	21	2.241	1.641	0.566	0.439	0.361	−0.170	100.00%	−0.191	0.312	0.374	2.210	0.000
*AHBZE*	30	2.172	1.552	0.506	0.342	0.320	−0.065	96.55%	−0.055	0.247	0.313	2.130	0.274
*AHBZF*	30	2.172	1.602	0.512	0.354	0.329	−0.056	96.55%	−0.060	0.249	0.310	2.170	0.433
*AHBZA*	30	2.207	1.553	0.491	0.386	0.312	−0.172	93.10%	−0.220	0.257	0.340	2.200	0.000
*AHBZB*	30	2.241	1.553	0.490	0.364	0.309	−0.144	89.66%	−0.163	0.256	0.345	2.240	0.002
*AHBZC*	29	2.103	1.545	0.472	0.319	0.298	−0.037	89.66%	−0.051	0.282	0.315	2.100	0.298
*AHBZD*	30	2.172	1.544	0.476	0.320	0.301	−0.037	93.10%	−0.046	0.279	0.329	2.140	0.611
*AHBZG*	28	2.483	1.596	0.532	0.286	0.322	0.128	93.10%	0.125	0.301	0.295	2.470	1.000
*AHBZH*	30	2.483	1.625	0.541	0.346	0.334	−0.012	96.55%	−0.020	0.288	0.287	2.460	0.999
*SDLCA*	30	2.448	1.589	0.520	0.320	0.317	−0.025	100.00%	0.005	0.283	0.298	2.410	1.000
*SDLCB*	28	2.207	1.548	0.485	0.322	0.309	−0.015	96.55%	−0.022	0.266	0.302	2.140	0.540
*SDHZE*	18	2.138	1.544	0.477	0.358	0.299	−0.129	89.66%	−0.175	0.269	0.334	2.090	0.080
*SDHZF*	19	2.138	1.589	0.504	0.354	0.320	−0.075	89.66%	−0.078	0.289	0.337	2.130	0.078
*SDHZB*	26	2.483	1.649	0.552	0.326	0.340	0.032	96.55%	0.048	0.311	0.349	2.120	0.996
*SDHZC*	26	2.207	1.673	0.541	0.336	0.343	−0.015	93.10%	0.032	0.296	0.344	2.170	0.961
*SDHZD*	27	2.345	1.663	0.537	0.308	0.336	0.043	93.10%	0.082	0.283	0.318	2.220	0.994
*SXMXA*	20	2.483	1.719	0.587	0.358	0.358	0.012	93.10%	0.022	0.362	0.347	2.420	0.958
*SXTG*	23	2.276	1.547	0.501	0.294	0.308	0.062	89.66%	0.080	0.269	0.338	2.210	1.000
*SXGQ*	16	2.483	1.679	0.570	0.324	0.346	0.052	89.66%	0.052	0.305	0.348	2.320	1.000
*SXMXB*	25	2.310	1.572	0.512	0.351	0.322	−0.070	100.00%	−0.060	0.290	0.313	2.240	0.075
*SXYS*	17	2.276	1.596	0.501	0.343	0.312	−0.061	86.21%	−0.052	0.262	0.346	2.250	0.397
*SXFX*	31	2.517	1.630	0.535	0.356	0.326	−0.063	96.55%	−0.044	0.276	0.415	2.280	0.697
*SXLFA*	29	2.345	1.606	0.536	0.370	0.335	−0.099	96.55%	−0.068	0.283	0.321	2.330	0.203
*SXLFB*	30	2.483	1.619	0.541	0.346	0.330	−0.031	100.00%	−0.039	0.295	0.388	2.440	0.994
*BJJF*	12	2.621	1.739	0.645	0.314	0.386	0.215	96.55%	0.080	0.335	0.317	1.810	1.000
*HBBK*	7	2.069	1.554	0.472	0.330	0.295	−0.111	82.76%	−0.051	0.246	0.356	2.000	0.403
*XXFH*	4	1.724	1.484	0.399	0.405	0.265	−0.471	65.52%	−0.087	0.237	0.369	1.610	0.013
*HNSY*	30	2.172	1.635	0.518	0.370	0.335	−0.088	93.10%	−0.093	0.275	0.356	2.140	0.101
*HNLY*	30	2.172	1.629	0.522	0.379	0.338	−0.099	96.55%	−0.088	0.286	0.340	2.130	0.230
*SXSN*	24	2.103	1.530	0.467	0.322	0.298	−0.016	93.10%	−0.123	0.260	0.332	2.510	0.058
*SXSLA*	29	2.103	1.596	0.495	0.348	0.316	−0.079	89.66%	−0.138	0.244	0.286	2.240	0.227
*SXSLB*	30	2.276	1.576	0.487	0.333	0.307	−0.030	89.66%	−0.055	0.301	0.359	2.250	0.995
*SDHZA*	20	2.034	1.496	0.430	0.288	0.268	−0.058	75.86%	−0.048	0.261	0.316	1.980	0.470
Total	25	2.259	1.595	0.513	0.343	0.321	−0.046	92.53%	−0.045	0.280	0.330	2.194	—

Note: *N* = Population size, *N*_*a*_ = Number of alleles per locus; *N*_*e*_ = Effective number of alleles; *I* = Shannon’s Information index; *H*_*O*_ = Observed heterozygosity; *H*_*E*_ = Expected heterozygosity; *F* = Wright’s fixation index; *PPL* = Percentage of Polymorphic Loci; *F*_*IS*_ = Inbreeding among individuals within subpopulations; *F*_*ST*_ = Genetic differentiation coefficient; *N*_*m*_ = Gene flow; *PIC* = Polymorphism information content; *H* = Genetic diversity; *A*_*R*_ = Allelic richness; *P*_HWE_ = *P* Value for HardyeWeinberg equilibrium.

The global multi-locus *F*_*ST*_ estimated for the 29 loci was 0.106 and ranged from 0.022 at locus PS260 to 0.529 at locus PS187. The pairwise multi-locus *F*_*ST*_ estimated for these loci ranged from 0.001 to 0.208, which indicated moderate population differentiation among the populations (Table S5).

### Population structure

**Figure 2:**
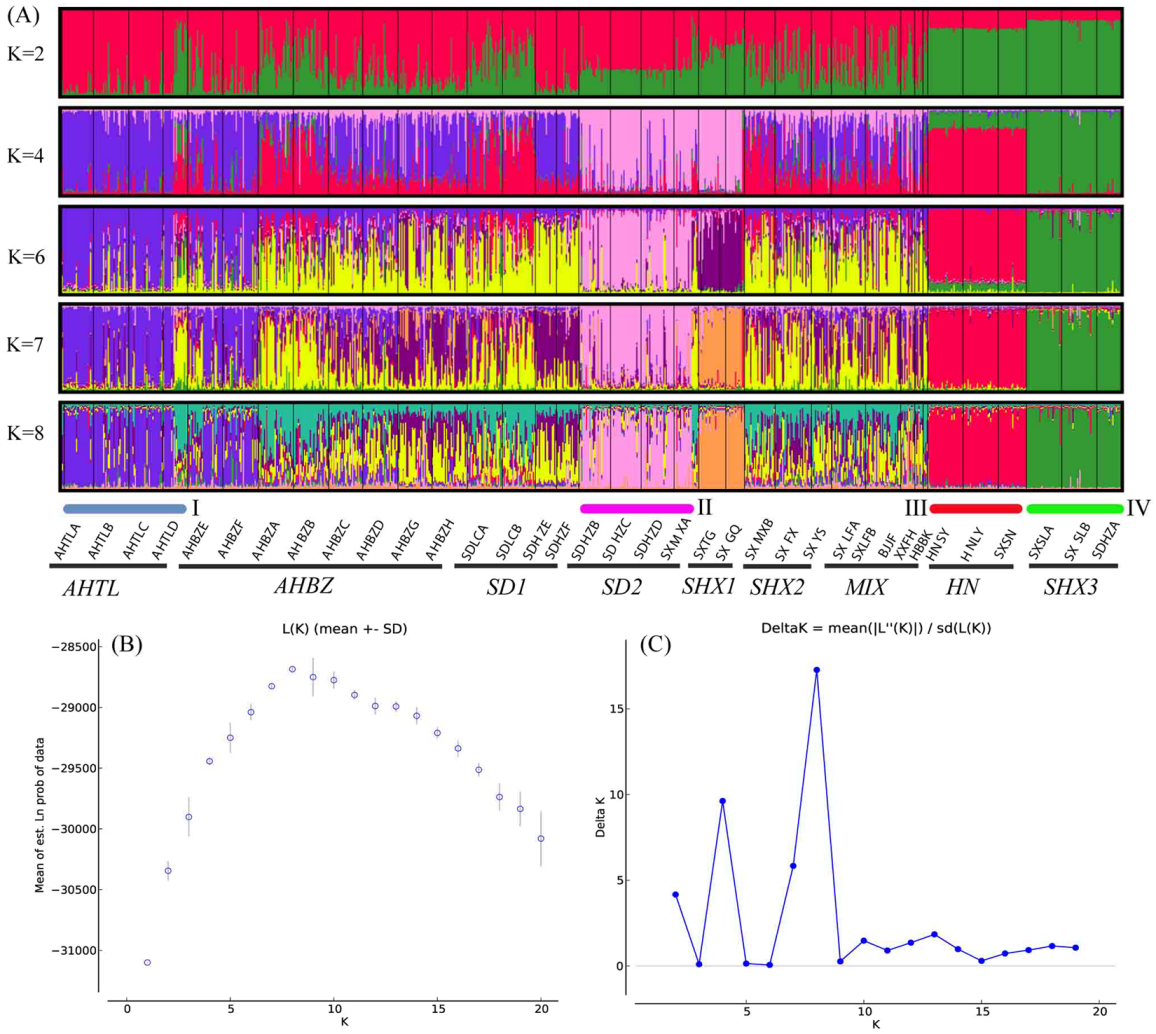
(**A**) Estimated population structure and clustering of the 901 *P. ostii* individuals with *K* = 2 to 8 (except *K* = 5). (**B**) Values of L(*K*) obtained in the STRUCTURE analyses. (**C**) *ΔK* estimates of the posterior probability distribution of the data for a given *K*.

STRUCTURE analyses indicated that the ln *P*(*D*) reached a clear mode at *K* = 8 before decreasing, and the highest delta *K* was detected when *K* = 8 with the second highest values were detected at *K* = 2, *K* = 4 and *K* = 7 (Fig. 2). The species genetic structure is discussed with the results up to *K* = 8 (except *K* = 5). Four major well-separated genetic clusters (I, II, III and IV) were identified at *K* = 4–8, and one minor well-separated genetic cluster (*SHX1*, composed of the *SXTG* and *SXGQ* populations) was observed at *K* = 6–8. Models assuming different *K* values helped to detect admixed groups (*AHBZ*, *SD1*, *SHX2* and *MIX*). Group (*AHTL*) of populations from Tongling in Anhui Province almost entirely made up cluster I; similarly, populations (*SDHZB*, *SDHZC*, *SDHZD*) from Shandong Province were always assigned to cluster II; three populations (*HNLY*, *SXSN* and *HNLY*) from Hunan, Shaanxi and Henan Province were assigned to cluster III; three populations (*SXSLA*, *SXSLB*, *SDHZA*) from Shaanxi and Shandong Province were assigned to cluster IV. These four major and one minor well-separated genetic clusters revealed the five gene pools in the domestication of the species, indicating probably five times of independent domestication events. Two populations (*AHBZE*, AHBZ*F*) from Bozhou City in Anhui Province showed a closer affiliation with cluster I at any *K* value, which may be attributed to recent migration from Tongling to Bozhou City in Anhui Province. When *K* = 4, the group (*AHBZ*) of populations from Bozhou City in Anhui Province, Linfen City in Shanxi Province (*SXLFA*, *SXLFB*) and Liaocheng City in Shandong Province (*SDLCA*, *SDLCB*) appeared to be admixed groups of clusters I and III, highlighting the important role of clusters I and III in recent breeding activities through hybridization. At *K* = 8, populations from Shaanxi Province were assigned to four different ancestral gene pools and populations from Shandong Province were assigned to two ancestral gene pools.

**Figure 3:**
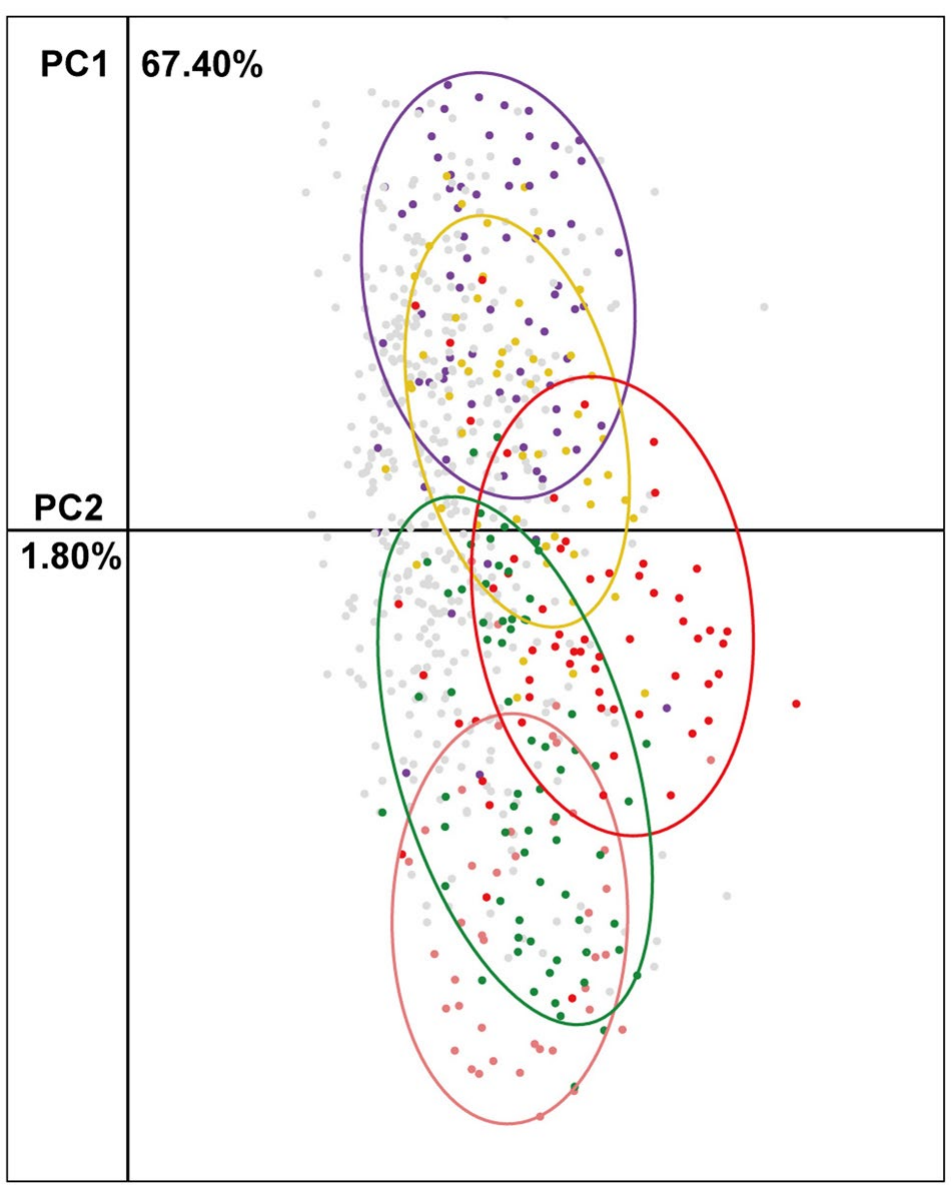
Principal coordinates analyses (PCAs) based on the matrix of *Nei*’s unbiased genetic distance among 901 sampled individuals.

A principal coordinate analysis (PCA) partitioned 67.40% and 1.80% of the variance in the experimental data to the first two axes (Fig. 3). Collectively, 69.2% of the variation is explained by the first two components. Although the PCA analysis showed a more continuous and overlapping pattern of genetic structure than the STRUCTURE analyses, the results were similar when the five ancestral gene pools were considered. The analyses of molecular variance (AMOVAs) revealed that most of the genetic diversity (93.77%) was observed within populations, whereas only 6.23% of the genetic variation occurred between the evaluated populations (Table 2). This observation could be attributed to the breeding system of the cross-pollinating plants.

**Table 2:** Analyses of molecular variance (AMOVAs) for the 36 *P. ostii* populations.

Source	d.f.	Sum of squares	Variance components	Percentage of variation	*P*-value
**1. Total**					
Among all population	35	958.667	0.421	6.23%	0.062***
Within Population	1768	11203.650	6.337	93.77%	
**2. Nine clustered grouped**					
Among regions	8	534.940	0.291	5.83%	0.07***
Among populations within regions	27	280.927	0.123	2.48%	
Within populations	1766	8082.679	4.571	91.70%	

Note: d.f. = degree of freedom; ***P < 0.001.

### Sample pooling and clustering

According to the results of the STRUCTURE analyses and the geographical sources of the samples, we divided our samples into nine groups to better identify the regional divergence among populations. Samples from the *HNSY*, *HNLY* and *SXSN* populations shared the same proportion of genetic ancestry in the STRUCTURE analyses; therefore, these samples were pooled into a single group (*HN*). Similarly, samples from the *SDHZB*, *SDHZC*, *SDHZD* and *SXMXA* populations were pooled together to form the *SD2* group, samples from the *SXTG* and *SXGQ* populations were pooled together to form the *SHX1* group, and samples from the *SXSLA*, *SXSLB* and *SDHZA* populations were pooled together to form the *SHX3* group. Samples collected from Tongling and Bozhou City in Anhui Province were pooled into the *AHTL* and *AHBZ* groups, respectively. Similarly, samples from Shandong and Shaanxi Provinces were pooled into the *SD1* and *SHX2* groups, respectively. Because a significant structure was not observed for certain samples, samples from *SXLFA*, *SXLFB*, *XXFH*, *HBBK* and *BJJF* were pooled into a single group (*MIX*). In total, nine groups were analyzed: *AHTL*, *AHBZ*, *SD1*, *SD2*, *SHX1*, *SHX2*, *MIX*, *HN* and *SHX3* (Fig. 2).

The global multi-locus *F*_*ST*_ for the groups was estimated at 0.076, and the pairwise multi-locus *F*_*ST*_ ranged from 0.001 to 0.170, indicating moderate genetic differentiation (Table 3). The *SHX3* group (from Shaanxi Province) showed the highest degree of genetic differentiation from the other groups (average *F*_*ST*_ = 0.130). Groups *SHX1* and *HN* (composed of samples from different geographically distant populations) showed moderate genetic differentiation compared with the other groups (average *F*_*ST*_ = 0.109–0.105). Group *AHTL* (the group of putatively original cultivation samples) and *SD2* showed low genetic differentiation compared with group *AHBZ*, *SD1*, *SHX2* and *MIX*, and this finding was consistent with the STRUCTURE analyses. The AMOVA analyses of the nine cluster groups revealed that 5.83% of the genetic variation occurred among regions, whereas 2.48% of the genetic variation occurred among populations within regions (Table 2).

**Table 3:** Pairwise multi-locus *F*_*ST*_ values for clustered samples.

	*AHTL*	*AHBZ*	*SD1*	*SD2*	*SHX1*	*SHX2*	*MIX*	*HN*	*SHX3*
*AHTL*	0	0.025	0.049	0.057	0.112	0.040	0.036	0.126	0.138
*AHBZ*	**0.000**	0	0.014	0.034	0.081	0.010	0.006	0.098	0.116
*SD1*	**0.000**	**0.000**	0	0.036	0.095	0.010	0.007	0.090	0.123
*SD2*	**0.000**	**0.000**	**0.000**	0	0.090	0.031	0.025	0.123	0.148
*SHX1*	**0.000**	**0.000**	**0.000**	**0.000**	0	0.082	0.076	0.137	0.170
*SHX2*	**0.000**	**0.000**	**0.000**	**0.000**	**0.000**	0	0.001	0.080	0.106
*MIX*	**0.000**	**0.000**	**0.000**	**0.000**	**0.000**	0.153	0	0.087	0.105
*HN*	**0.000**	**0.000**	**0.000**	**0.000**	**0.000**	**0.000**	**0.000**	0	0.134
*SHX3*	**0.000**	**0.000**	**0.000**	**0.000**	**0.000**	**0.000**	**0.000**	**0.000**	0

Note: *F*_*ST*_ values are above the diagonal and associated *P-*values below. All *P*-values in bold were judged significant after sequential Bonferroni correction.

**Figure 4:**
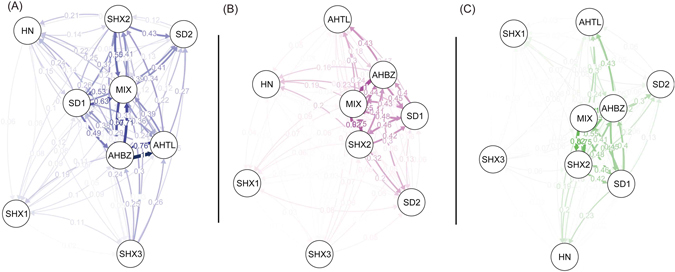
Directional relative migration networks of clustered samples from divMigrates. (**A**) Jost’s *D* values, (**B**) *G*_*ST*_ values and (**C**) *N*_*m*_ values.

The directional relative migration networks as estimated by divMigrate^53^ depicted the relative migration rates among the nine groups (Fig. 4a–c). For all three estimators (Jost’s *D*, *G*_*ST*_, and *Nm*), clusters were grouped in a pattern similar to that of the STRUCTURE analyses and pairwise *F*_*ST*_ analyses. Four admixed groups (*AHBZ*, *SD1*, *SHX2* and *MIX*) that grouped close together displayed a high degree of gene flow, and two genetically pure groups (*AHTL* and *SD2*) exhibited a relatively high gene flow with these four admixed groups. Three genetically distinct groups (*SHX1*, *SHX3* and *HN*) appeared relatively isolated from the other groups.

## Discussion

To establish breeding and management strategies for emerging crops, a thorough understanding of the population genetics of the species of interest has become essential for producing long-term sustainable goals. This work represents the first systematic population genetic study of *P. ostii*, and we selected unlinked and neutral markers and performed range-wide genotyping and genetic evaluation for this commercially important woody plant with a long history of cultivation.

### Genetic diversity and future exploration

*P. ostii* presents a wide distribution range, reproduces via insect pollination and outcrossing and has a self-incompatible mating system, and these characteristics allow this species to develop a higher genetic diversity. Consistent with other cross-pollinating plants^54, 55^, most of the total genetic variation (>90%) in *P. ostii* occurs among individuals within a population, whereas a small proportion of the genetic variation occurs between populations. Compared with previous studies of wild peony species in the *Moutan* section, the genetic diversity indices obtained in the present study are lower than those in *Paeonia jishanensis* ^56^ (*Na* = 4.762, *Ho* = 0.446, *He* = 0.340) and *Paeonia rockii* ^57^ (*Na* = 3.69, *Ho* = 0.459, *He* = 0.492). This could be attributed to long-term domestication of *P. ostii* involving selecting and modifying its wild progenitor to meet human needs^58^. The elite *P. ostii* cultivars that growers use today are the result of intensive selection applied to the founding stock and its descendants, which is presumed to have led to additional losses in genetic diversity. The reduction in genetic diversity does not bode well for future genetic gains in productivity and could result in broad susceptibility to newly emerging diseases or insect pests, thereby threatening long-term food and feed security^59, 60^. Future germplasm collections for the creation of new cultivars should account for the vast reservoir of genetic diversity to maintain the genetic base of *P. ostii*.

STRUCTURE analyses identified five main ancestral gene pools across the wide range of *P. ostii* samples. Interestingly, we found that samples in Shaanxi Province were assigned to four ancestral gene pools. In addition, the genetic diversity analyses demonstrated that both the diversity and allelic richness are generally higher in populations from Shaanxi Province. Based on these results, we proposed that Shaanxi Province is the main genetic diversity center of the cultivated materials of the species. The other important group of populations from Tongling (*AHTL*) in Anhui Province contained a well-separated gene pool and displayed extensive genetic exchanges with admixed groups as revealed by the divMigrate analyses. Therefore, the resources from these two provinces could represent priorities for future germplasm conservation and exploration.

### Population structure and domestication history

Cultivation of *P. ostii* for medicinal use originally occurred in Shaanxi, Henan, Shandong and Anhui (Tongling) provinces^29, 61^. The hypothesis that the domestication origin of *P. ostii* occurred in Shaanxi Province has generally been accepted, although other hypotheses of its domestic origin are still popular^61^. However, the domestic origin hypotheses have not been tested using genetic data. The present study presented for the first time a genetic test for *P. ostii* based on an analysis of multi-locus data. Five genetically well-separated gene pools were identified in geographically isolated regions of Shaanxi Province, Tongling City in Anhui Province, and in Shandong, Henan and Hunan provinces. The allopatric distribution of distinct gene pools of cultivated *P. ostii* provided support for the hypothesis of independent domestication origins of this species. Consistent with the cultivated ornamental tree peony^62^, our study provided another case of multiple independent domestication for the cultivated tree peonies of the *Moutan* section.

In the present study, the STRUCTURE results of five well-separated gene pools indicated that five independent domestication origins may have occurred over the long-term cultivation of *P. ostii*. Interestingly, four distinct gene pools were found in Shaanxi Province, with two of these pools also found in Shandong. The richest diversity of both the wild populations and species of tree peony was observed in Shaanxi^25, 26^, and this province also presents the longest recorded history of cultivation of tree peonies^63–65^. Reports have suggested that the tree peony was introduced from Shaanxi to Shandong Province several different times from ancient to modern times^28, 63, 65^. Given these facts, it is easy to predict that at least four times domestication origins of cultivated *P. ostii* ever happened in Shaanxi Province. The reconstruction of four well-separated gene pools identified by the STRUCTURE analyses clearly suggested that protection and utilization should be prioritized for the cultivars of different domestication origins that are isolated from each other in areas such as *SXSN* (Shangnan, Shaanxi Province), *SXMXA* (Meixian, Shaanxi Province), *SXSLA* (Shangnan, Shaanxi Province), *SXSLB* (Shangzhou, Shaanxi Province), *SXGQ* (Ganquan, Shaanxi Province), and *SXTG* (Tongguan, Shaanxi Province). Because most of these areas are located in the vicinity of the Qinling mountains, which presents the largest specific diversity of the *Moutan* section, we suspect that the Qinling Mountains in Shaanxi represents the likely origin of domesticated *P. ostii*. As the wild plants of *P. ostii* have been completely eradicated^33^ and all populations sampled from Shaanxi were cultivated, further analysis of *P. ostii*, including models of the domestication history and phylogenetic reconstructions based on the low-copy nuclear genes relative to that of the chloroplast and rDNA sequences, will be needed to validate this hypothesis.

As the world’s largest producer of ornamental tree peony, Heze City in Shandong Province cultivates *P. ostii* resources that are extensively used as rootstock for ornamental cultivated tree peony grafting. Our present study suggested that at least two rounds of independent long-distance *P. ostii* introductions likely occurred from Shaanxi to Shandong. We observed that one well-separated gene pool was identified in three distinct populations of *SXSN* (Shangnan, Shaanxi), *HNLY* (Luoyang, Henan Province) and *HNSY* (Shaoyang, Hunan Province), reflected probably unique domestication in Shaanxi Province and following long-distance introduction to Henan and Hunan provinces. It is not difficult to see the importance of Shaanxi Province in domestication and deployment of *P. ostii*.

Another important region in the domestication of *P. ostii* is Tongling City in Anhui Province, where the long-term cultivation of this species has been recorded^27^. We found a group (*AHTL*) of populations from Tongling that almost solely consisted of a unique well-separated gene pool (cluster I). This finding indicates at least one independent domestication origin in Tongling City, which is distinct from the origins in Shaanxi Province. It is interesting that all cultivated samples from Tongling City were assigned to one unique gene pool without introduction were found from Shaanxi Province.

Our study suggested that Tongling City has likely played an important role in the domestication and breeding of modern cultivars of *P. ostii*. Bozhou City, which is located close to Tongling City, has earned a reputation as the “Capital of Traditional Herbs” and has become the central region for the cultivation of *P. ostii* ^66^. The production of *P. ostii* in Bozhou accounts for more than 70% of the annual total production^29, 66^. In our present study, both the STRUCTURE and divMigrate analyses revealed that cultivars in Bozhou City and admixed cultivars popular in other regions are similar in genetic makeup and present the closest relationships with cultivars from Tongling City in Anhui Province, Shaanxi and Shandong provinces. This finding demonstrates the important role of domestication in Tongling City in Anhui Province.

Alongside with those from well-separated gene pools, admixed individuals were identified extensively across most of the sampled regions (Shaanxi, Shandong, Beijing, Shanxi, Anhui, Hubei and Hunan). We hypothesized that these individuals were likely derived from recent hybridization breeding processes, but we failed to find records that corroborated this hypothesis. Genomic data may help elucidate the origin of the admixed populations.

## Conclusions

This study was the first comprehensive work to select well-characterized SSR markers for population genetics studies on peony species. Our study settled long-running debates by confirming that (1) moderate genetic diversity and differentiation in *P. ostii*; (2) Shaanxi Province has the largest genetic diversity; (3) multiple independent domestication origins occurred in Shaanxi Province and Tongling City in Anhui Province, and this species was introduced to Shandong, Henan and Hunan provinces. Our results provide comprehensive information that can be used in genetic assessments, regional genetic improvements and management of *P. ostii* and insights for the development of breeding strategies for this emerging oil crop.

## Materials and Methods

### Sample collection for the population genetic study

**Figure 5:**
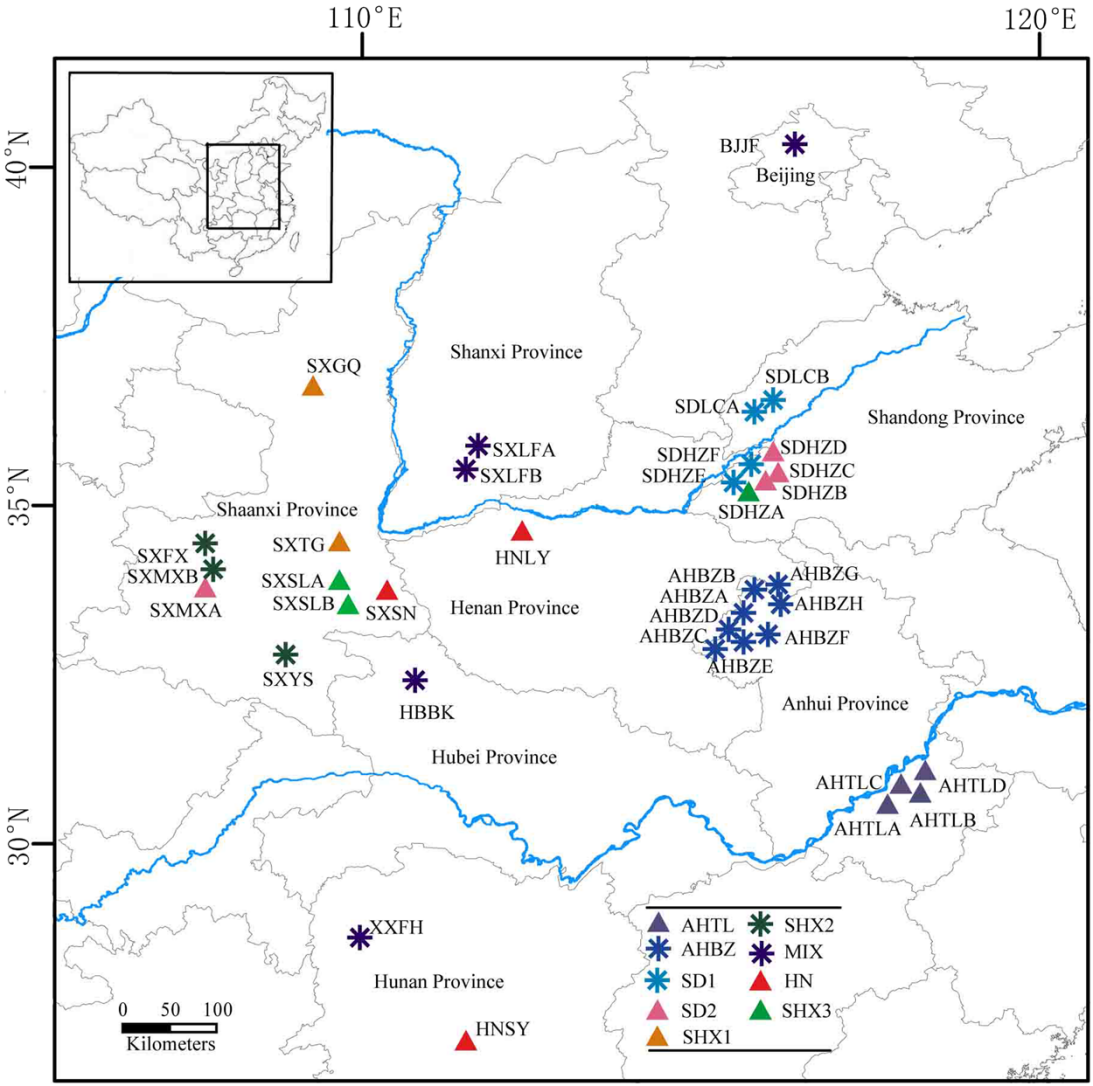
Geographical distribution of the 36 sampled *P. ostii* populations. Figure 5 was created in ArcGIS 10.0 http://www.esrichina.com.cn/.

A total of 902 individuals from 36 populations that cover the vast majority of the distribution area in China were collected (Fig. 5). The geographical distribution of the 36 sampled populations was shown in Supplementary Table S1. Young leaves were collected in the field individually, dried with colored silica gel^67^, and maintained at room temperature for DNA extraction.

### SSR development and genetic mapping

A total of 59,275 unigenes were obtained from the transcriptome assembly of *P. suffruticosa* ‘Luo Yang Hong’^48^, and 4,373 potential SSRs were identified from 3,787 unigene sequences. In total, 2,989 SSR-containing sequences were appropriate for primer design, and 788 primer pairs were further selected for the primer synthesis. Of these, 373 primer pairs generated PCR amplification products of the expected sizes^43^. Those 373 SSR loci were then screened for polymorphism in a genetic mapping population. The mapping population was composed of 197 individuals, including the two parents and 195 F_1_ progeny. The parents were *P. ostii* ‘FenDanBai’ (female parent) and *P. × suffruticosa* ‘HongQiao’ (male parent). The F_1_ mapping population plants were grown in the Beijing Guose Peony Garden, Beijing, China. The validity and polymorphism of 373 SSR loci were analyzed by using 6 progeny individuals randomly selected from F_1_ progeny, and 74 SSR loci were found to be polymorphic. We kept these 74 SSR loci for genotyping of 195 mapping individuals. All SSR genotypes we generated were combined with SNP genotypes^68^ from the same mapping population, and then used for chisquare test (χ^2^) of segregation and genetic map construction with Joinmap 4.1. Fifty-three SSR loci showed no deviation from Mendelian inheritance ratios (*P* < 0.05). Then, the optimal arrangement order was determined via regression analyses with the single-linkage clustering algorithm at logarithm of odds (LOD) ≥ 4.0 and recombination rates (*r*) ≤ 0.3. Based on the above results, the LOD threshold was relaxed to 3.0 and markers that showed segregation distortion with higher LOD values in the linkage analyses were also mapped to the linkage group without changing the order of marker linearity. The genetic distance between adjacent markers was then estimated. The graph of the genetic map with 4 SNP markers (Marker19470, Marker3373, Marker18459 and Marker36271) and 68 SSR markers was visualized using MapChart v2.3^69^.

To further examine the genetic diversity and structural analyses of the 902* P. ostii* individuals, 68 SSR loci on the genetic linkage map (Fig. 1) were used to perform polymorphism screening in random selected 8 *P. ostii* individuals and 40 polymorphic markers evenly distributed all 5 linkage groups (LGs) were selected.

### DNA extraction and genotyping

Total genomic DNA was extracted from 30 mg of silica-dried leaf material using the DNeasy Plant Genomic DNA Kit (Qiagen, Beijing, China). The purity and concentration of the extracted genomic DNA was measured using a NanoDrop 2000 spectrophotometer at 260 nm (Thermo Scientific, USA) and adjusted to 50 ng/μL. PCR amplification was performed in a total volume of 10 μL containing 1 μL (50 ng) genomic DNA, 3 μL ddH_2_O, 5 μL 1 × Taq PCR Master Mix (Aide Lai, Beijing, China), and 0.5 μL (10 pmol) each reverse and forward primer with fluorescent labeled 6-FAM, HEX, TAMRA or ROX (Ruiboingke, Beijing, China). PCR amplification was performed with the following amplification protocol: pre-denaturation at 95 °C for 4 min, followed by 35 cycles of denaturation at 94 °C for 35 s, annealing at 47–60 °C for 35 s (different primer annealing temperatures are shown in Supplementary Table S1) and extension at 72 °C for 35 s, with a final extension at 72 °C for 7 min. The PCR products were analyzed separately with an ABI 3730XL capillary sequencer along with an internal size standard (GeneScan-500 LIZ, Applied Biosystems). The SSR allele sizes were scored manually using GeneMarker v2.2 (Soft Genetics, State College, Pennsylvania, USA) for all populations.

### Statistical analyses

#### Suitability of microsatellites for the population genetic analyses

We further checked the suitability of each marker for the population genetic analyses. Micro-Checker was used to identify possible genotyping errors, including stuttering, large allele drop-outs and null alleles. We determined whether selected polymorphic microsatellite markers deviated significantly from a neutral equilibrium model using Ewen-Watterson tests in Arlequin.

#### Genetic diversity indices and genetic differentiation

The number of alleles per locus (*N*), number of different alleles (*N*_*a*_), number of effective alleles (*N*_*e*_), Shannon’s information index (*I*), observed heterozygosity (*H*_*O*_), expected heterozygosity (*H*_*E*_ *)*, percentage of polymorphic loci (*PPL*), and F-statistics (*F*_*IS*_, *F*_*ST*_) were calculated using GeneAlEx v6.501^70, 71^. The polymorphism information content (*PIC*) was calculated with PowerMarker v3.25^72^. Hardy–Weinberg equilibrium (HWE) was calculated with the assistance of Genepop (http://genepop.curtin.edu.au/). Samples with identical genotypes were detected by GeneCap.

#### Genetic structure

The model-based (Bayesian) cluster software STRUCTURE v2.3.4^73^ was chosen to estimate the population structure. For each value of *K* (*K* = 1–20), ten independent runs were performed with a burn-in period of 200,000 followed by 200,000 Markov Chain Monte Carlo (MCMC) replications. The most likely *K* value was determined using Structure Harvester^74^ based on both the log likelihood and the maximum *ΔK*. Because clustering algorithms may incorporate stochastic simulations as part of the inference, independent analyses of the same data may result in several distinct outcomes; therefore, we used the computer program CLUMPP v1.1.2^75^ to analyze the results from replicate analyses for optimal alignments of replicate clusters. The output from CLUMPP was graphically displayed by the cluster visualization program DISTRUCT^76^.

A PCA based on the pairwise *F*_*ST*_ distance matrix was performed using the “adegenet” package^77^ in R^78^.

#### Pairwise genetic distance and gene flow among pooled collections

To identify the genetic differences in the 901 *P. ostii* samples, sampled populations were pooled according to their geographic origin and genetic composition, and the global multi-locus *F*_*ST*_ for the 29 loci was calculated with GenAlEx. The pairwise multi-locus *F*_*ST*_ and significance of differences between *F*_*ST*_ values was assessed in exact tests conducted with Arlequin, and the gene flow (*N*_*m*_) based on the *F*_*ST*_ values was calculated as *N*_*m*_ = 0.25 (1 − *F*_*ST*_)/*F*_*ST*_. A genetic distance matrix of pairwise *FST* values was also used for AMOVAs^79^ in Arlequin. Significance levels were determined using 1000 permutations.

## Electronic supplementary material

Supplement Table
