# Supplementary material for: Genetic analyses reveal independent domestication origins of the emerging oil crop Paeonia ostii, a tree peony with a long-term cultivation history
Source: Sci Rep. 2017 Jul 13;7:5340. doi: 10.1038/s41598-017-04744-z (PMC5509724; doi:10.1038/s41598-017-04744-z)
Supplement: Supplementary file 1 — Supplement Table [file 41598_2017_4744_MOESM1_ESM.doc]

**Genetic analyses reveal independent domestication origins of the emerging oil crop *Paeonia ostii*, a tree peony with a long-term cultivation history**

Li-Ping Peng^1^, Chang-Fu Cai^1^, Yuan Zhong ^1^, Xing-Xing Xu^1^, Hong-Li Xian^2^, Fang-Yun Cheng^1^,*, Jian-Feng Mao^1^,*

^1^ National Flower Engineering Research Centre, College of Landscape Architecture, National Engineering Laboratory for Tree Breeding, Key Laboratory of Genetics and Breeding in Forest Trees and Ornamental Plants, Ministry of Education, College of Biological Sciences and Technology, Beijing Forestry University, Beijing, China

^2^ Forestry Department of Shaanxi Province, Xian, China

* **Correspondence to:**

Dr. Fang-Yun Cheng (E-mail: chengfy8@263.net)

Dr. Jian-Feng Mao (E-mail: jianfeng.mao@bjfu.edu.cn)

**Table S1 Geographical location of analyzed *P.ostii* populations in this study.**

**Table S2 Characterization of 74 SSR markers.**

**Table S3 Locus level genetic diversity estimates for 901 individuals of *P. ostii.***

**Table S4 Summary of private alleles by population.**

**Table S5 Pairwise multi-locus *F_ST_* for all samples with the 29 loci.**

**Table S1 Geographical location of analyzed *P.ostii* populations in this study.**

| **Code** | **Location of population** | **AS^1^** | **NS^2^** | **Latitude**  **(°**N**)** | **Longtitude**  **(°E)** | **Altitude**  **(m)** | **Remarks** |
| --- | --- | --- | --- | --- | --- | --- | --- |
|
|
| *AHTLA* | RuiPu peony material base, Tongling city, Anhui Province | 6 | 27 | 30.8712 | 118.0218 | 85 | cultivated |
| *AHTLB* | Fenghuang mountain, Tongling city, Anhui Province | 9 | 30 | 30.8820 | 118.0200 | 105 | cultivated |
| *AHTLC* | Fenghuang mountain, Tongling city, Anhui Province | 6 | 29 | 30.8818 | 118.0224 | 98 | cultivated |
| *AHTLD* | JinFeng Village, Zhongming town, Tongling city, Anhui Province | 10 | 21 | 30.9142 | 118.0752 | 151 | cultivated |
| *AHBZE* | Ligezi viliage, Wuma town, Bozhou city, Anhui Province | 9 | 30 | 33.8775 | 115.8826 | 34 | cultivated |
| *AHBZF* | Ligezi viliage, Wuma town, Bozhou city, Anhui Province | - | 30 | 33.8740 | 115.8795 | 34 | cultivated |
| *AHBZA* | Shijiuli town, Qiaocheng District, Bozhou city, Anhui Province | 6 | 30 | 33.7824 | 115.8499 | 38 | cultivated |
| *AHBZB* | Shijiuli town, Qiaocheng District, Bozhou city, Anhui Province | 7 | 30 | 33.7779 | 115.8490 | 37 | cultivated |
| *AHBZC* | Shibali town, Qiaocheng District, Bozhou city, Anhui Province | 6 | 30 | 33.8551 | 115.6664 | 42 | cultivated |
| *AHBZD* | Shibali town, Qiaocheng District, Bozhou city, Anhui Province | 5 | 30 | 33.8661 | 115.6721 | 40 | cultivated |
| *AHBZG* | Shibali town, Qiaocheng District, Bozhou city, Anhui Province | 7 | 29 | 33.8657 | 115.6743 | 37 | cultivated |
| *AHBZH* | Wangxingzhuang viliage, Huatuo town, Bozhou city, Anhui Province | 12+ | 30 | 34.6114 | 116.3146 | 38 | cultivated |
| *SDLCA* | Mei Garden, DongE county, Liaocheng city, Shandong Province | 8 | 30 | 36.3530 | 116.2841 | 34 | cultivated |
| *SDLCB* | Mei Gaden, DongE county, Liaocheng city, Shandong Province | 9 | 28 | 36.3342 | 116.3062 | 34 | cultivated |
| *SDHZE* | Pili Village, Peony District, Heze city, Shandong Province | 9+ | 18 | 35.4308 | 115.9249 | 35 | cultivated |
| *SDHZF* | Pili Village, Peony District, Heze city, Shandong Province | 8+ | 19 | 35.4120 | 115.9033 | 34 | cultivated |
| *SDHZB* | Xiaoliu town, Peony District, Heze city, Shandong Province | 5 | 27 | 35.6123 | 115.7426 | 33 | cultivated |
| *SDHZC* | HuangGang town, Peony District, Heze city, Shandong Province | 15+ | 26 | 35.8007 | 116.2622 | 35 | cultivated |
| *SDHZD* | NiJi town, Cao county, Heze city, Shandong Province | 3 | 28 | 36.4339 | 115.8343 | 38 | cultivated |
| *SXMXA* | Dali Village, YingKou Town, Mei county, Baoji city, Shaanxi Province | - | 21 | 34.1334 | 107.8978 | - | putative natural population |
| *SXTG* | ZhoujiaCheng village, TongGuan county, Shangluo city, Shaanxi Province | 13 | 23 | 34.1600 | 110.2536 | - | cultivated |
| *SXGQ* | Guanquan county, Yanan city, ShaanXi Province | 15 | 16 | 36.7362 | 109.1657 | 965 | cultivated |
| *SXMXB* | Dali Village, YingKou Town, Mei county, Baoji city, Shaanxi Province | 7 | 26 | 34.1599 | 107.7583 | - | cultivated |
| *SXYSA* | YangShan Village, Gouyuan Town, Xunyang city, Shaanxi Province | - | 17 | 32.7899 | 109.5861 | - | cultivated |
| *SXFX* | Miganqiao Town, Fengxiang country, Baoji city, Shaanxi Province | 8 | 31 | 34.1603 | 107.7652 | - | cultivated |
| *SXLFA* | Yuan Village, Nantang Town, Linfen city, ShanXi Province | 7 | 29 | 35.6752 | 111.6409 | 546 | cultivated |
| *SXLFB* | XixiaPing Village, Nantang Town, Linfen city, ShanXi Province | 5 | 30 | 35.6594 | 111.6278 | 538 | cultivated |
| *BJJF* | Jiufeng National Forest Garden, Beijing city | 9+ | 12 | 40.0630 | 116.0978 | 127 | research base |
| *HBBK* | Houping Villiage Baokang county, Xiangyang city, Hubei Province | - | 9 | 31.7197 | 113.6867 | 34 | cultivated |
| *XXFH* | FengHuang city, Tujia and Miao Autonomous Prefecture, Hunan Province | 15+ | 4 | 28.5932 | 110.0025 | 48 | cultivated |
| *HNSY* | LijiaPing town, Shaoyang City, Hunan Province | 7 | 30 | 27.0165 | 111.6151 | - | cultivated |
| *HNLY* | National peony Garden, Luoyang City, Henan Province | 15+ | 30 | 34.7068 | 112.3929 | 177 | cultivated in garden |
| *SXSN* | Shangnan county, Shangluo city, Shaanxi Province | - | 24 | 33.8163 | 110.6281 | 608 | putative natural population |
| *SXSLA* | Shangzhou District, Shangluo city, Shaanxi Province | - | 30 | 33.8651 | 109.9353 | - | cultivated |
| *SXSLB* | Shangzhou District, Shangluo city, Shaanxi Province | 7 | 30 | 33.8471 | 109.9044 | - | cultivated |
| *SDHZA* | Xiaoliu town, Peony District, Heze city, Shandong Province | 9 | 20 | 35.6020 | 115.7353 | 33 | cultivated |

**Note:** ^1^Age of samples; ^2^Number of samples

**Table S2 Characterization of 74 SSR markers.**

| **Primer ID** | **Genetic Distance**  **(cM)** | **Linkage Group** | **Primer Sequence (5'-3')** | **Primer Sequence (3'-5')** | **T_m_**  **(℃)** | **Product size**  **(bp)** | **Motif** | **Segregation type** | **Medel Segregation ratio** | **Separation ratio** | **χ^2^ value** | **d.f.** |  |
| --- | --- | --- | --- | --- | --- | --- | --- | --- | --- | --- | --- | --- | --- |
| PS180 | 29.951 | LG1 | CCCCGAAATGGAGGAGTC | CCTAGAACCGTTAGCAACT | 60 | 188 | (CT)_6_ | <nn×np> | 1:1 | 84:111 | 3.74 | 1 |  |
| PS276* | 42.436 | LG1 | CTGTATCCTATCGGTTCTT | ACCTCTTTCTTGCTGCTC | 52.5 | 447 | (CCGGTG)_4_ | <lm×ll> | 1:1 | 150:45 | 56.54 | 1 |  |
| PS321 | 51.386 | LG1 | TGCTACCAGCTCCTCCAT | ACGGGCTCCTTACCACAT | 53 | 249 | (ATCA)_5_ | <nn×np> | 1:1 | 108:87 | 2.26 | 1 |  |
| PS337* | 69.217 | LG1 | ATCCTCTTCACGGCAATC | CATCTCCTCCTCAAACAT | 53.5 | 448 | (ACTGCC)_3_ | <ef×eg> | 1:1:1:1 | 53:35:54:53 | 5.18 | 3 |  |
| PS318* | 77.136 | LG1 | ATCTACTCCCTATCCGTCAC | CTGTTGTTCCTCCCACCT | 55 | 369 | (CCACA)_4_ | <ab×cd> | 1:1:1:1 | 35:52:57:51 | 5.59 | 3 |  |
| PS089*^#^ | 87.03 | LG1 | ACTGGACTGAAGGTGGAT | AACTCCTCCTCGTTGTCT | 56.5 | 333 | (ATA)_6_ | <ef×eg> | 1:1:1:1 | 53:46:38:58 | 4.65 | 3 |  |
| PS257*^#^ | 89.148 | LG1 | GGCAAAAGAACGAGAAAAC | TCGCCAACTTCTTCTTTC | 54 | 234 | (CTCGTA)_4_ | <nn×np> | 1:1 | 96:99 | 0.05 | 1 |  |
| PS026*^#^ | 105.978 | LG1 | TTCCCTCCATTCTAACAC | ACCCTAGCCTCTGACATT | 54 | 187 | (AG)_6_ | <nn×np> | 1:1 | 123:72 | 13.34 | 1 |  |
| PS339 | 110.177 | LG1 | TGAGGCAGCCAAAGAATT | CTCCTCTTTAACTCCTCCAT | 50 | 175 | (ACAGCA)_3_ | <nn×np> | 1:1 | 58:137 | 32.01 | 1 |  |
| PS159 | 134.196 | LG1 | CCTCCATTCATTCCTGTC | CGGCAAAATAATAAGTAAAGAG | 52 | 124 | (CT)_7_ | <nn×np> | 1:1 | 91:104 | 0.87 | 1 |  |
| PS205* | 134.196 | LG1 | ATTCAAATAGTCTTCGTGC | TGTCTGTCTCATCGTCCC | 55 | 316 | (AAAT)_4_ | <lm×ll> | 1:1 | 91:104 | 0.87 | 1 |  |
| PS221*^#^ | 173.579 | LG1 | GATACAAGGCGGAAAGTG | ATGGGTCGGTAAACAACAG | 56 | 301 | (AAT)_5_ | <nn×np> | 1:1 | 79:116 | 7.02 | 1 |  |
| PS108* | 206.757 | LG1 | GCTAAGGCGGTCTTCAGG | TGTTTCCCTGTTGTCCTC | 58.5 | 434 | (CAA)_5_ | <nn×np> | 1:1 | 93:102 | 0.42 | 1 |  |
| PS074*^#^ | 207.987 | LG1 | TGCCTTGCTCCTCCTTGT | ACTGCTGTGGGCATAGGG | 57 | 236 | (CT)_7_ | <nn×np> | 1:1 | 93:102 | 0.42 | 1 |  |
| PS273*^#^ | 212.892 | LG1 | CCCTCAGATGGGATGGAA | GGTGCCCAATTCAGGTAG | 58.5 | 314 | (GCCGCT)_4_ | <nn×np> | 1:1 | 93:102 | 0.42 | 1 |  |
| PS345 | 219.257 | LG1 | TGAAGTGAATCGAAGCAT | CATTGGTAGGCGGTCTTT | 48 | 366 | (TGGTTG)_3_ | <nn×np> | 1:1 | 94:101 | 0.25 | 1 |  |
| PS271*^#^ | 0 | LG2 | AGAATCCACCTCCTGTCAC | GATTCTTTCTGTTGTGGG | 56.5 | 406 | (GGAGAA)_3_ | <lm×ll> | 1:1 | 86:109 | 2.71 | 1 |  |
| PS351 | 2.015 | LG2 | GGGTTTGATTAGGCTTCG | CTACCACCATAAGTGTAAGAG | 56.5 | 324 | (GGATCA)_3_ | <nn×np> | 1:1 | 90:105 | 1.15 | 1 |  |
| PS242 | 40.128 | LG2 | TCTAAGCCAGAGCCCAAGT | ATTGTTTGGTCGTCCTTT | 57.5 | 199 | (TCTCGT)_3_ | <nn×np> | 1:1 | 89:106 | 1.48 | 1 |  |
| PS299 | 61.053 | LG2 | ATCTAACACCCTTCCTCC | CACCTGAACTCACCCTCC | 54 | 261 | (CATCTC)_3_ | <hk×hk> | 1:2:1 | 47:108:40 | 2.76 | 2 |  |
| PS336 | 69.218 | LG2 | GACCACGGTGAGCTTATT | CTCCAACCATTTGTCCCT | 50 | 339 | (ACCCAT)_3_ | <nn×np> | 1:1 | 100:95 | 0.13 | 1 |  |
| PS073 | 87.281 | LG2 | GTCGGTGAATGAAGGGTT | ACATTTCAGCCAACACTC | 53.5 | 269 | (AG)_6_ | <nn×np> | 1:1 | 95:100 | 0.13 | 1 |  |
| PS263 | 96.25 | LG2 | TAACCCACAACCCACTTC | CTCCCACTCGTCAATGCT | 55 | 327 | (TCACCA)_3_ | <ef×eg> | 1:1:1:1 | 44:57:47:47 | 1.98 | 3 |  |
| PS119*^#^ | 100.157 | LG2 | GCAAAGACAACAGCCTCG | ATCCAAACAGAAAGTATGAG | 57 | 289 | (CAG)_6_ | <ef×eg> | 1:1:1:1 | 59:44:47:45 | 2.97 | 3 |  |
| PS365*^#^ | 104.089 | LG2 | AACCAAACTAACCCTAAATG | GTTGGAGTTGGAGAGGTTG | 49 | 350 | (CAA)_7_ | <nn×np> | 1:1 | 95:100 | 0.13 | 1 |  |
| PS101*^#^ | 120.065 | LG2 | AAACCCTTACTTCCACTT | CCCTACATAACAGACGAAAAG | 51 | 153 | (CTA)_8_ | <nn×np> | 1:1 | 108:87 | 2.26 | 1 |  |
| PS162*^#^ | 124.166 | LG2 | CAGTCTTTCATGGCTTCAC | AAGCAACACCGACAGAGG | 51 | 357 | (AG)_6_ | <nn×np> | 1:1 | 79:116 | 7.02 | 1 |  |
| PS112*^#^ | 128.334 | LG2 | TCCAAATACACGCTCGTT | ATCTCCTGCGACCTTATC | 54 | 320 | (ACC)_5_ | <lm×ll> | 1:1 | 94:101 | 0.25 | 1 |  |
| PS047 | 129.706 | LG2 | AGACGACGAGCAAAGATAT | CGATGCGTAAATGTCAAAT | 54 | 126 | (TC)_8_ | <nn×np> | 1:1 | 68:127 | 17.85 | 1 |  |
| PS004* | 179.158 | LG2 | GTGCTTAGCCTCTAATCTG | CTTTGCTCCAAGTCTGTC | 50.5 | 274 | (GA)_8_ | <ef×eg> | 1:1:1:1 | 49:53:42:51 | 1.41 | 3 |  |
| PS029*^#^ | 185.679 | LG2 | CAACTTGTTCTCCGTCCTT | GCAGGCGATGCTACAAAC | 54 | 304 | (GA)_7_ | <ef×eg> | 1:1:1:1 | 52:57:32:54 | 7.93 | 3 |  |
| PS265*^#^ | 187.771 | LG2 | TTTTATGGGTCCTGTTGC | GACAACCTTGCCTAGAAT | 54 | 290 | (ACAGCC)_4_ | <hk×hk> | 1:2:1 | 52:93:50 | 0.46 | 2 |  |
| PS356*^#^ | 215.101 | LG2 | TCAAGCCCAAGGTCATTC | TGCTATGGTAAGCGGAAA | 53 | 354 | (GAT)_7_ | <ef×eg> | 1:1:1:1 | 34:42:59:60 | 10.15 | 3 |  |
| PS139 | 215.883 | LG2 | CAACAATTTAACACGCAGAG | ATCTCGGCTCAAATCGTC | 56.5 | 482 | (CCG)_6_ | <nn×np> | 1:1 | 77:118 | 8.62 | 1 |  |
| PS309*^#^ | 223.047 | LG2 | AAGCAAAGCCGTGGAGAT | ACCATCTCCACCATTTCG | 55 | 257 | (CT)_6_ | <nn×np> | 1:1 | 81:114 | 5.58 | 1 |  |
| PS296 | 306.845 | LG2 | CTCTTTCGCTGCCACAAC | GTCTTATGGCGGCTATGT | 57.5 | 419 | (GAAGCA)_4_ | <lm×ll> | 1:1 | 85:110 | 3.21 | 1 |  |
| PS106* | 0 | LG3 | GGCTGAACTTCTATCACTTT | CCCATCAACCCATAATCC | 53.5 | 202 | (ACC)_6_ | <nn×np> | 1:1 | 86:109 | 2.71 | 1 |  |
| PS149*^#^ | 1.563 | LG3 | AGTCGCCTCCTACACCTC | CTCAACAACCCTATCACAT | 55.5 | 173 | (AGG)_5_ | <nn×np> | 1:1 | 94:101 | 0.25 | 1 |  |
| PS335 | 18.683 | LG3 | TAATCACCCAATGAGCCA | CCTCATCAGGGTTCTCAC | 50 | 395 | (TTCATT)_3_ | <nn×np> | 1:1 | 83:112 | 4.31 | 1 |  |
| PS061*^#^ | 26.013 | LG3 | CTCCTCCAACATTGACCC | ATTTCTGGTCAATGTGGC | 57 | 154 | (TG)_8_ | <nn×np> | 1:1 | 85:110 | 3.21 | 1 |  |
| PS158* | 36.732 | LG3 | TTTCCCTGCTTCTTCTGAC | TGTTAGTGATGAAATGGGAT | 55 | 423 | (CT)_7_ | <nn×np> | 1:1 | 110:85 | 3.21 | 1 |  |
| PS068* | 38.129 | LG3 | CTTTGGCATTCTCATTCA | ACCCTCGTGTCTGAGTGCTAC | 52.5 | 174 | (TC)_7_ | <nn×np> | 1:1 | 84:111 | 3.74 | 1 |  |
| PS199* | 41.905 | LG3 | ACAAAAGGAAATGAGAAGCG | CTGTTGATCGTTGGGTTT | 55 | 351 | (GA)_6_ | <nn×np> | 1:1 | 86:109 | 2.71 | 1 |  |
| PS323 | 70.221 | LG3 | CTCACCCGTTCTAAAGTCA | CGTCGTCGCCGAATACTT | 53 | 466 | (CGATTC)_3_ | <nn×np> | 1:1 | 106:89 | 1.48 | 1 |  |
| PS260*^#^ | 70.221 | LG3 | ATTCACGCCAGTATCAAAG | CCCTTACTCGCCATAGCAC | 53 | 349 | (CCTGGA)_3_ | <nn×np> | 1:1 | 91:104 | 0.87 | 1 |  |
| PS045 | 78.434 | LG3 | CGGAGTGGTGGATAGTGT | TAACATCCAAAGCAACTC | 52 | 110 | (GA)_6_ | <nn×np> | 1:1 | 86:109 | 2.71 | 1 |  |
| PS066 | 81.664 | LG3 | AAGAACATCAAGGGGAGA | CGTCCCTTTCTGTATCCTT | 51.5 | 183 | (TG)_7_ | <nn×np> | 1:1 | 89:106 | 1.48 | 1 |  |
| PS367*^#^ | 0 | LG4 | AGACGGACGGAAATAGGG | AAATACTCCGTCTTAGTGTGAA | 53.5 | 265 | (TCC)_7_ | <nn×np> | 1:1 | 111:84 | 3.74 | 1 |  |
| PS146 | 15.294 | LG4 | GACCACCTCCTCCCAATC | CACCTCCTTCCTTTCTTACT | 57 | 117 | (TCT)_5_ | <nn×np> | 1:1 | 109:86 | 2.71 | 1 |  |
| PS095 | 15.294 | LG4 | TCCCAAGACCTCAAACAAC | AATGGATGATGGGAGTGC | 55 | 394 | (CCA)_5_ | <nn×np> | 1:1 | 111:84 | 3.74 | 1 |  |
| PS329 | 18.299 | LG4 | ATCTTTCGTTGGCTTCCT | GATGCCGTTCATGCTCCC | 56 | 408 | (GCCTTC)_3_ | <lm×ll> | 1:1 | 105:90 | 1.15 | 1 |  |
| PS249*^#^ | 46.854 | LG4 | GGTTCTGCTCTAGGGTCG | TTTCATCGGAAAGGACAAC | 57.5 | 303 | (GGTGGA)_3_ | <hk×hk> | 1:2:1 | 48:109:38 | 3.74 | 2 |  |
| PS333*^#^ | 50.019 | LG4 | AAGCACGGTGTTCCAGAT | CAACAGGCAGAAGAAAGG | 52.5 | 415 | (TCACCA)_3_ | <lm×ll> | 1:1 | 85:110 | 3.21 | 1 |  |
| PS157 | 68.574 | LG4 | CTCCCTGAACTCCCTACC | AAAGGGAAGAAGAGTGAG | 56 | 322 | (AG)_6_ | <nn×np> | 1:1 | 108:87 | 2.26 | 1 |  |
| PS290*^#^ | 82.911 | LG4 | TTCTTTCACCTCCACTTCA | GATGTCCCGTCGCTTGTG | 53 | 245 | (AGTAGG)_4_ | <lm×ll> | 1:1 | 111:84 | 3.74 | 1 |  |
| PS033 | 99.188 | LG4 | AAGGAATAGAGTTGTTGGGATG | CTTTCGCTTACTGGTTGA | 54.5 | 323 | (CT)_7_ | <nn×np> | 1:1 | 65:130 | 21.67 | 1 |  |
| PS006* | 0 | LG5 | TTGTTCTGTTCAAGGGTT | TTCTTATCCAGCCGAAAG | 54.5 | 217 | (AT)_6_ | <nn×np> | 1:1 | 113:82 | 4.93 | 1 |  |
| PS311*^#^ | 8.353 | LG5 | AACGCCACCATCACCTTT | CCTCCTCCCTGTTCTTCT | 60 | 277 | (TTC)_6_ | <nn×np> | 1:1 | 89:106 | 1.48 | 1 |  |
| PS076*^#^ | 9.915 | LG5 | ATGCCACCTTTTCCTAAT | AGACGGTGATAGTGAGGA | 48.5 | 258 | (TC)_8_ | <nn×np> | 1:1 | 88:107 | 1.85 | 1 |  |
| PS183*^#^ | 18.878 | LG5 | CACTGTAGGTGCCTTGTAAC | CTTCTTCCTGGGCTGTGAG | 52 | 144 | (CT)_6_ | <nn×np> | 1:1 | 101:94 | 0.25 | 1 |  |
| PS369 | 39.562 | LG5 | CACAAGTTGGGTAGAAAGA | TATACTCAGCCTCGAAAAGAA G | 55.5 | 251 | (CCA)_5_ | <nn×np> | 1:1 | 87:108 | 2.26 | 1 |  |
| PS187*^#^ | 52.348 | LG5 | AAGCGGCGTCCATCATAC | TGAACCATCGTGGGAAGC | 57 | 233 | (GA)_7_ | <nn×np> | 1:1 | 74:121 | 11.33 | 1 |  |
| PS014 | 86.034 | LG5 | ATGGAAAGTATTGTGGGC | AAAAGGAAGGGGAAGAAAAC | 51 | 358 | (GT)_6_ | <nn×np> | 1:1 | 125:70 | 15.51 | 1 |  |
| PS134*^#^ | 109.379 | LG5 | CATCAACTTCGGCTAACA | AGAAGCAAGCCACGAATG | 55 | 387 | (CAC)_5_ | <ef×eg> | 1:1:1:1 | 38:40:47:70 | 13.27 | 3 |  |
| PS166* | 116.803 | LG5 | TTCAGTGGGCAAGACCTAC | GGGCGAGAAGAAGGAGAAG | 55 | 337 | (AT)_7_ | <nn×np> | 1:1 | 75:120 | 10.38 | 1 |  |
| PS030 | 162.386 | LG5 | ACCCTCCACCACCATCTT | CGCGAAACAAGGATGGAG | 57 | 237 | (CT)_7_ | <nn×np> | 1:1 | 140:55 | 37.05 | 1 |  |
| PS069 | 194.037 | LG5 | GAGAAAGGAGGAGGGAGG | ATGATTCCGAGGAGGGTG | 54.5 | 128 | (GA)_6_ | <nn×np> | 1:1 | 163:32 | 88.01 | 1 |  |
| PS144*^#^ | 275.638 | LG5 | CAACCTACAATCCGACAATG | TGTGGGTAGTGGTTTGTTAG | 54.5 | 317 | (TGC)_5_ | <ef×eg> | 1:1:1:1 | 37:74:36:46 | 18.52 | 3 |  |
| PS085 | - | - | CACGGTAGGGAACAACTT | CCCATGACTCGTCGTGCT | 53.5 | 450 | (TGA)_5_ | <ef×eg> | 1:1:1:1 | 70:75:24:26 | 46.58 | 3 |  |
| PS111 | - | - | GCAGATCATGGCGACAAAAC | TTACCTCCATCTCATACG | 57.5 | 372 | (CAA)_5_ | <lm×ll> | 1:1:1:1 | 94:101 | 0.25 | 1 |  |
| PS210 | - | - | CCACCAGATACATGCCACA | ACAGTGCGTGGTTGTGCT | 56.5 | 289 | (ACAA)_4_ | <ef×eg> | 1:1:1:1 | 31:26:68:67 | 28.9 | 3 |  |
| PS270 | - | - | CCCCACATACCCAAATCG | GAGACTCGGAACAAGGAAG | 57.5 | 253 | (CCTCTT)_3_ | <nn×np> | 1:1 | 99:96 | 0.05 | 1 |  |
| PS284 | - | - | GCATAGAGGAATAAGGAGGAT | CTCTGCTCTTCCCGTCTT | 55.5 | 496 | (TGGAAA)_3_ | <nn×np> | 1:1 | 145:50 | 46.28 | 1 |  |
| PS252 | - | - | CATTCCCAACCCTCCAAC | TCTCGGCGGGTTTCTTAT | 58.5 | 493 | (TTCACC)_3_ | <ef×eg> | 1:1:1:1 | 41:50:49:55 | 2.07 | 3 |  |
| **Note:** *locus showing polymorphism in *P. ostii* populations; ^#^locus used for genetic evaluation in *P. ostii.* Mendelian inheritance ratios *P*<0.05 (d.f.=1, χ^2^=3.84; d.f.=2, χ^2^=5.99; df=3, χ^2^=7.81), d.f.=degree of freedom | | | | | | | | | | | | |  |

| **Table S3 Locus level genetic diversity estimates for 901 individuals of *P. ostii.*** | | | | | | | | | | | | | |
| --- | --- | --- | --- | --- | --- | --- | --- | --- | --- | --- | --- | --- | --- |
| **Locus** | **N** | ***N_a_*** | ***N_e_*** | ***I*** | ***H_O_*** | ***H_E_*** | ***F*** | ***F_IS_*** | ***F_ST_*** | ***N_m_*** | ***H*** | ***PIC*** | ***P*_HWE_** |
| **PS101** | 3 | 2.056 | 1.889 | 0.662 | 0.801 | 0.464 | -0.694 | -0.724 | 0.040 | 5.936 | 0.482 | 0.368 | 0.000 |
| **PS026** | 2 | 1.972 | 1.930 | 0.663 | 0.536 | 0.475 | -0.129 | -0.129 | 0.049 | 4.824 | 0.508 | 0.386 | 0.009 |
| **PS290** | 4 | 2.056 | 1.452 | 0.477 | 0.307 | 0.300 | -0.005 | -0.021 | 0.036 | 6.786 | 0.314 | 0.268 | 0.821 |
| **PS311** | 5 | 1.917 | 1.543 | 0.489 | 0.437 | 0.325 | -0.321 | -0.345 | 0.079 | 2.927 | 0.364 | 0.303 | 0.000 |
| **PS273** | 5 | 2.583 | 1.306 | 0.419 | 0.212 | 0.222 | 0.043 | 0.045 | 0.036 | 6.712 | 0.222 | 0.209 | 0.996 |
| **PS356** | 5 | 2.139 | 1.385 | 0.440 | 0.238 | 0.267 | 0.051 | 0.107 | 0.036 | 6.747 | 0.274 | 0.238 | 1.000 |
| **PS061** | 5 | 2.889 | 2.098 | 0.803 | 0.567 | 0.520 | -0.091 | -0.091 | 0.034 | 7.081 | 0.538 | 0.431 | 0.072 |
| **PS257** | 2 | 1.944 | 1.172 | 0.261 | 0.123 | 0.141 | 0.105 | 0.129 | 0.026 | 9.199 | 0.148 | 0.137 | 1.000 |
| **PS029** | 7 | 3.000 | 2.185 | 0.872 | 0.548 | 0.536 | -0.013 | -0.022 | 0.111 | 1.996 | 0.602 | 0.526 | 0.994 |
| **PS260** | 3 | 1.889 | 1.118 | 0.200 | 0.091 | 0.102 | 0.052 | 0.106 | 0.022 | 11.056 | 0.113 | 0.108 | 0.992 |
| **PS144** | 5 | 2.278 | 1.924 | 0.696 | 0.454 | 0.474 | 0.036 | 0.042 | 0.069 | 3.353 | 0.522 | 0.414 | 0.954 |
| **PS162** | 5 | 2.167 | 1.542 | 0.538 | 0.309 | 0.340 | 0.077 | 0.091 | 0.026 | 9.367 | 0.378 | 0.329 | 1.000 |
| **PS074** | 4 | 1.417 | 1.141 | 0.128 | 0.080 | 0.082 | 0.007 | 0.032 | 0.442 | 0.315 | 0.171 | 0.159 | 0.676 |
| **PS249** | 5 | 2.250 | 1.932 | 0.693 | 0.524 | 0.479 | -0.094 | -0.096 | 0.039 | 6.167 | 0.505 | 0.391 | 1.000 |
| **PS265** | 6 | 2.111 | 1.925 | 0.680 | 0.469 | 0.476 | 0.009 | 0.013 | 0.053 | 4.462 | 0.512 | 0.396 | 1.000 |
| **PS089** | 3 | 1.972 | 1.299 | 0.374 | 0.236 | 0.222 | -0.064 | -0.062 | 0.027 | 8.919 | 0.259 | 0.234 | 0.081 |
| **PS333** | 5 | 2.583 | 1.899 | 0.699 | 0.470 | 0.461 | -0.021 | -0.020 | 0.052 | 4.592 | 0.499 | 0.401 | 0.623 |
| **PS149** | 6 | 2.139 | 1.171 | 0.270 | 0.146 | 0.140 | -0.053 | -0.042 | 0.025 | 9.752 | 0.150 | 0.141 | 0.296 |
| **PS187** | 5 | 2.639 | 1.299 | 0.385 | 0.212 | 0.203 | -0.031 | -0.045 | 0.529 | 0.223 | 0.448 | 0.414 | 0.994 |
| **PS271** | 5 | 2.639 | 1.820 | 0.648 | 0.403 | 0.408 | 0.016 | 0.013 | 0.114 | 1.953 | 0.492 | 0.419 | 0.994 |
| **PS309** | 7 | 2.667 | 1.596 | 0.534 | 0.378 | 0.311 | -0.130 | -0.217 | 0.258 | 0.719 | 0.443 | 0.403 | 0.005 |
| **PS365** | 5 | 2.028 | 1.141 | 0.223 | 0.111 | 0.115 | 0.021 | 0.032 | 0.045 | 5.349 | 0.155 | 0.149 | 0.995 |
| **PS183** | 3 | 1.472 | 1.135 | 0.133 | 0.120 | 0.081 | -0.197 | -0.495 | 0.302 | 0.578 | 0.114 | 0.108 | 0.000 |
| **PS367** | 8 | 2.278 | 1.808 | 0.656 | 0.450 | 0.432 | -0.014 | -0.041 | 0.157 | 1.341 | 0.514 | 0.444 | 0.997 |
| **PS112** | 7 | 3.444 | 2.712 | 1.077 | 0.637 | 0.625 | -0.019 | -0.019 | 0.031 | 7.892 | 0.670 | 0.613 | 0.996 |
| **PS076** | 3 | 2.444 | 1.415 | 0.464 | 0.243 | 0.268 | 0.063 | 0.090 | 0.159 | 1.327 | 0.329 | 0.306 | 0.999 |
| **PS221** | 8 | 2.167 | 1.364 | 0.343 | 0.147 | 0.188 | 0.097 | 0.222 | 0.189 | 1.076 | 0.334 | 0.315 | 1.000 |
| **PS134** | 7 | 2.333 | 1.389 | 0.463 | 0.262 | 0.269 | 0.021 | 0.026 | 0.031 | 7.767 | 0.282 | 0.252 | 1.000 |
| **PS119** | 4 | 2.028 | 1.674 | 0.580 | 0.431 | 0.391 | -0.083 | -0.101 | 0.051 | 4.626 | 0.445 | 0.371 | 0.302 |
| **Total** | 142 | 2.259 | 1.595 | 0.513 | 0.343 | 0.321 | -0.046 | -0.053 | 0.106 | 4.932 | 0.372 | 0.318 | - |
| Note: N = Number of alleles per locus; *N_a_* = Number of Different Alleles; *N_e_* = Effective number of alleles; *I* = Shannon’s Information index; *H_O_* = Observed heterozygosity; *H_E_* = Expected heterozygosity; *F* = Wright’s fixation index; *F_IS_* = Inbreeding among individuals within subpopulations; *F_ST_* = Genetic differentiation coefficient; *N_m_* = Gene flow; *H* = Gene diversity; *PIC* = Polymorphism information content; *P*_HWE_= *P* Value for Hardy Weinberg equilibrium. | | | | | | | | | | | | | |

| **Table S4 Summary of private alleles by population.** | | | |
| --- | --- | --- | --- |
| **Population** | **Locus** | **Allele** | **Frequence** |
| *AHTLB* | PS221 | 258 | 0.056 |
| *AHBZA* | PS074 | 238 | 0.017 |
| *SXLFB* | PS290 | 186 | 0.033 |
| *SXLFB* | PS273 | 274 | 0.033 |
| *SXLFB* | PS265 | 303 | 0.033 |
| *SXYS* | PS356 | 348 | 0.029 |
| *SXYS* | PS089 | 329 | 0.029 |
| *SXMXC* | PS271 | 336 | 0.032 |
| *SXMXC* | PS309 | 267 | 0.016 |
| *HBBK* | PS367 | 253 | 0.071 |
| *HBBK* | PS221 | 321 | 0.071 |
| *AHBZG* | PS311 | 232 | 0.069 |
| *AHBZG* | PS311 | 270 | 0.069 |
| *AHBZG* | PS365 | 301 | 0.038 |
| *AHBZG* | PS365 | 305 | 0.038 |
| *AHBZH* | PS162 | 263 | 0.037 |
| *AHBZH* | PS119 | 394 | 0.033 |
| *SDHZE* | PS265 | 368 | 0.056 |
| *SDLCA* | PS162 | 257 | 0.034 |
| *BJJF* | PS273 | 313 | 0.083 |
| *BJJF* | PS265 | 378 | 0.083 |
| *BJJF* | PS149 | 168 | 0.125 |
| *BJJF* | PS183 | 145 | 0.042 |
| *BJJF* | PS119 | 285 | 0.083 |
| *SXGQ* | PS311 | 277 | 0.031 |
| *SXGQ* | PS029 | 306 | 0.063 |
| *SXGQ* | PS144 | 254 | 0.063 |
| *SXGQ* | PS149 | 184 | 0.031 |
| *SXGQ* | PS367 | 271 | 0.031 |
| *SXGQ* | PS112 | 309 | 0.036 |
| *SXGQ* | PS134 | 388 | 0.094 |
| *SDHZB* | PS260 | 310 | 0.037 |
| *SDHZB* | PS367 | 213 | 0.038 |
| *SDHZC* | PS112 | 320 | 0.038 |
| *SDHZD* | PS221 | 323 | 0.033 |
| *SDHZA* | PS290 | 227 | 0.025 |
| *SXSLA* | PS265 | 236 | 0.038 |
| *SXSLB* | PS221 | 312 | 0.017 |
| *SXSLB* | PS134 | 400 | 0.067 |

| **Table S5 Pairwise multi-locus *F_ST_* for all samples with the 29 loci** | | | | | | | | | | | | | | | | | | | | | | | | | | | | | | | | | | | | |
| --- | --- | --- | --- | --- | --- | --- | --- | --- | --- | --- | --- | --- | --- | --- | --- | --- | --- | --- | --- | --- | --- | --- | --- | --- | --- | --- | --- | --- | --- | --- | --- | --- | --- | --- | --- | --- |
|  | AHTLA | AHTLB | AHTLC | AHTLD | AHBZE | AHBZF | AHBZA | AHBZB | AHBZC | AHBZD | AHBZG | AHBZH | SDLCA | SDLCB | SDHZE | SDHZF | SDHZB | SDHZC | SDHZD | SXMXA | SXTG | SXGQ | SXMXB | SXFX | SXYS | SXLFA | SXLFB | BJJF | HBBK | XXFH | HNSY | HNLY | SXSN | SXSLA | SXSLB | SDHZA |
| AHTLA | 0 | 0.001 | 0.001 | 0.047 | 0.066 | 0.068 | 0.039 | 0.067 | 0.082 | 0.056 | 0.084 | 0.059 | 0.057 | 0.053 | 0.010 | 0.008 | 0.083 | 0.053 | 0.071 | 0.064 | 0.151 | 0.125 | 0.153 | 0.076 | 0.084 | 0.085 | 0.059 | 0.136 | 0.150 | 0.070 | 0.081 | 0.080 | 0.063 | 0.182 | 0.176 | 0.161 |
| AHTLB | 0.793 | 0 | 0 | 0.026 | 0.052 | 0.058 | 0.026 | 0.050 | 0.062 | 0.041 | 0.039 | 0.066 | 0.036 | 0.003 | 0.004 | 3E-05 | 0.056 | 0.032 | 0.051 | 0.040 | 0.134 | 0.111 | 0.136 | 0.056 | 0.068 | 0.067 | 0.032 | 0.115 | 0.130 | 0.046 | 0.059 | 0.060 | 0.040 | 0.164 | 0.157 | 0.150 |
| AHTLC | 0.847 | 0.766 | 0 | 0.047 | 0.076 | 0.081 | 0.049 | 0.067 | 0.076 | 0.059 | 0.070 | 0.079 | 0.055 | 0.005 | 0.021 | 0.012 | 0.081 | 0.053 | 0.078 | 0.064 | 0.152 | 0.131 | 0.156 | 0.081 | 0.086 | 0.087 | 0.058 | 0.130 | 0.142 | 0.069 | 0.076 | 0.075 | 0.061 | 0.183 | 0.178 | 0.166 |
| AHTLD | **1E-05** | **1E-05** | **1E-05** | 0 | 0.024 | 0.036 | 0.028 | 0.043 | 0.046 | 0.004 | 0.009 | 0.072 | 0.041 | 0.045 | 0.039 | 0.023 | 0.037 | 0.048 | 0.056 | 0.030 | 0.108 | 0.091 | 0.115 | 0.053 | 0.048 | 0.047 | 0.022 | 0.114 | 0.109 | 0.036 | 0.053 | 0.068 | 0.050 | 0.118 | 0.106 | 0.100 |
| AHBZE | **1E-05** | **1E-05** | **1E-05** | **1E-05** | 0 | 0.001 | 0.018 | 0.035 | 0.068 | 0.026 | 0.039 | 0.022 | 0.032 | 0.041 | 0.040 | 0.045 | 0.057 | 0.045 | 0.060 | 0.041 | 0.093 | 0.078 | 0.093 | 0.034 | 0.028 | 0.035 | 0.036 | 0.104 | 0.093 | 0.050 | 0.070 | 0.081 | 0.044 | 0.135 | 0.131 | 0.132 |
| AHBZF | **1E-05** | **1E-05** | **1E-05** | **1E-05** | 0.604 | 0 | 0.019 | 0.036 | 0.063 | 0.030 | 0.060 | 0.016 | 0.040 | 0.044 | 0.039 | 0.051 | 0.063 | 0.045 | 0.066 | 0.047 | 0.100 | 0.082 | 0.102 | 0.033 | 0.026 | 0.023 | 0.040 | 0.100 | 0.103 | 0.052 | 0.073 | 0.081 | 0.047 | 0.142 | 0.132 | 0.131 |
| AHBZA | **1E-05** | **1E-05** | **1E-05** | **1E-05** | **1E-05** | **1E-05** | 0 | 0.009 | 0.043 | 0.001 | 0.029 | 0.023 | 0.016 | 0.012 | 0.007 | 0.013 | 0.040 | 0.013 | 0.034 | 0.017 | 0.101 | 0.082 | 0.107 | 0.026 | 0.029 | 0.026 | 0.008 | 0.092 | 0.096 | 0.023 | 0.035 | 0.036 | 0.013 | 0.132 | 0.117 | 0.116 |
| AHBZB | **1E-05** | **1E-05** | **1E-05** | **1E-05** | **1E-05** | **1E-05** | 0.036 | 0 | 0.052 | 0.006 | 0.044 | 0.028 | 0.028 | 0.028 | 0.029 | 0.035 | 0.041 | 0.030 | 0.037 | 0.036 | 0.092 | 0.077 | 0.098 | 0.006 | 0.004 | 0.005 | 0.010 | 0.090 | 0.104 | 0.030 | 0.032 | 0.040 | 0.015 | 0.129 | 0.126 | 0.131 |
| AHBZC | **1E-05** | **1E-05** | **1E-05** | **1E-05** | **1E-05** | **1E-05** | **1E-05** | **1E-05** | 0 | 0.046 | 0.037 | 0.008 | 0.036 | 0.043 | 0.063 | 0.054 | 0.045 | 0.048 | 0.082 | 0.051 | 0.117 | 0.102 | 0.146 | 0.074 | 0.079 | 0.062 | 0.043 | 0.098 | 0.102 | 0.055 | 0.062 | 0.072 | 0.057 | 0.142 | 0.144 | 0.117 |
| AHBZD | **1E-05** | **1E-05** | **1E-05** | **1E-05** | **1E-05** | **1E-05** | 0.279 | 0.072 | **1E-05** | 0 | 0.042 | 0.018 | 0.019 | 0.022 | 0.017 | 0.033 | 0.045 | 0.026 | 0.047 | 0.034 | 0.099 | 0.080 | 0.101 | 0.029 | 0.029 | 0.034 | 0.019 | 0.098 | 0.101 | 0.035 | 0.052 | 0.052 | 0.020 | 0.120 | 0.110 | 0.117 |
| AHBZG | **1E-05** | **1E-05** | **1E-05** | 0.144 | **1E-05** | **1E-05** | 0.045 | **1E-05** | 0.045 | **1E-05** | 0 | 0.131 | 3E-04 | 0.004 | 0.062 | 0.034 | 0.032 | 0.030 | 0.049 | 0.013 | 0.129 | 0.112 | 0.141 | 0.007 | 0.067 | 0.064 | 0.002 | 0.093 | 0.094 | 0.034 | 0.039 | 0.058 | 0.055 | 0.176 | 0.142 | 0.140 |
| AHBZH | **0.009** | **1E-05** | **1E-05** | **0.009** | **1E-05** | 0.063 | 0.063 | 0.027 | **0.009** | 0.09 | **1E-05** | 0 | 0.065 | 0.072 | 0.042 | 0.054 | 0.083 | 0.047 | 0.070 | 0.065 | 0.097 | 0.072 | 0.088 | 0.044 | 0.025 | 0.053 | 0.049 | 0.119 | 0.114 | 0.065 | 0.093 | 0.098 | 0.052 | 0.115 | 0.125 | 0.104 |
| SDLCA | **1E-05** | **1E-05** | **1E-05** | **1E-05** | **1E-05** | **1E-05** | **1E-05** | **1E-05** | **1E-05** | **1E-05** | 0.396 | **1E-05** | 0 | 0.001 | 0.029 | 0.029 | 0.045 | 0.016 | 0.040 | 0.021 | 0.122 | 0.100 | 0.132 | 0.061 | 0.056 | 0.054 | 0.013 | 0.083 | 0.101 | 0.039 | 0.040 | 0.053 | 0.047 | 0.150 | 0.139 | 0.134 |
| SDLCB | **1E-05** | **1E-05** | **1E-05** | **1E-05** | **1E-05** | **1E-05** | **0.009** | **1E-05** | **1E-05** | **1E-05** | 0.288 | **1E-05** | 0.982 | 0 | 0.024 | 0.022 | 0.045 | 0.010 | 0.046 | 0.021 | 0.119 | 0.096 | 0.126 | 0.056 | 0.053 | 0.047 | 0.011 | 0.083 | 0.102 | 0.038 | 0.040 | 0.048 | 0.039 | 0.164 | 0.140 | 0.138 |
| SDHZE | **0.009** | 0.144 | **1E-05** | **1E-05** | **1E-05** | **1E-05** | 0.054 | **1E-05** | **1E-05** | **1E-05** | **1E-05** | 0.018 | **1E-05** | **1E-05** | 0 | 0.008 | 0.058 | 0.021 | 0.045 | 0.041 | 0.116 | 0.094 | 0.121 | 0.033 | 0.045 | 0.042 | 0.018 | 0.121 | 0.127 | 0.040 | 0.054 | 0.049 | 0.028 | 0.148 | 0.138 | 0.134 |
| SDHZF | **0.045** | 0.405 | **0.009** | **1E-05** | **1E-05** | **1E-05** | **0.009** | **1E-05** | **1E-05** | **1E-05** | **0.009** | **0.009** | **1E-05** | **1E-05** | **1E-05** | 0 | 0.042 | 0.024 | 0.035 | 0.029 | 0.120 | 0.101 | 0.128 | 0.043 | 0.051 | 0.051 | 0.025 | 0.105 | 0.124 | 0.043 | 0.044 | 0.046 | 0.035 | 0.166 | 0.138 | 0.132 |
| SDHZB | **1E-05** | **1E-05** | **1E-05** | **1E-05** | **1E-05** | **1E-05** | **1E-05** | **1E-05** | **1E-05** | **1E-05** | 0.018 | **1E-05** | **1E-05** | **1E-05** | **1E-05** | **1E-05** | 0 | 0.014 | 0.017 | 1E-05 | 0.137 | 0.119 | 0.145 | 0.056 | 0.059 | 0.056 | 0.034 | 0.106 | 0.136 | 0.045 | 0.057 | 0.061 | 0.050 | 0.172 | 0.160 | 0.157 |
| SDHZC | **1E-05** | **1E-05** | **1E-05** | **1E-05** | **1E-05** | **1E-05** | 0.018 | **1E-05** | **1E-05** | **1E-05** | 0.018 | 0.018 | **1E-05** | 0.036 | **1E-05** | **1E-05** | 0.027 | 0 | 0.014 | 0.000 | 0.121 | 0.101 | 0.131 | 0.042 | 0.050 | 0.046 | 0.011 | 0.096 | 0.113 | 0.030 | 0.039 | 0.038 | 0.029 | 0.176 | 0.144 | 0.149 |
| SDHZD | **1E-05** | **1E-05** | **1E-05** | **1E-05** | **1E-05** | **1E-05** | **1E-05** | **1E-05** | **1E-05** | **1E-05** | **1E-05** | **0.009** | **1E-05** | **1E-05** | **1E-05** | **1E-05** | **1E-05** | 0.018 | 0 | 6E-04 | 0.141 | 0.119 | 0.144 | 0.042 | 0.047 | 0.053 | 0.022 | 0.121 | 0.149 | 0.047 | 0.053 | 0.059 | 0.058 | 0.178 | 0.171 | 0.158 |
| SXMXA | **1E-05** | **1E-05** | **1E-05** | **1E-05** | **1E-05** | **1E-05** | **1E-05** | **1E-05** | **1E-05** | **1E-05** | 0.108 | **1E-05** | **1E-05** | **1E-05** | **1E-05** | **1E-05** | 0.468 | 0.73 | 0.261 | 0 | 0.133 | 0.112 | 0.139 | 0.05 | 0.055 | 0.053 | 0.012 | 0.103 | 0.118 | 0.033 | 0.036 | 0.045 | 0.043 | 0.175 | 0.156 | 0.153 |
| SXTG | **1E-05** | **1E-05** | **1E-05** | **1E-05** | **1E-05** | **1E-05** | **1E-05** | **1E-05** | **1E-05** | **1E-05** | **1E-05** | **1E-05** | **1E-05** | **1E-05** | **1E-05** | **1E-05** | **1E-05** | **1E-05** | **1E-05** | **1E-05** | 0 | 0.001 | 0.012 | 0.076 | 0.083 | 0.087 | 0.081 | 0.161 | 0.128 | 0.129 | 0.135 | 0.145 | 0.096 | 0.163 | 0.131 | 0.147 |
| SXGQ | **1E-05** | **1E-05** | **1E-05** | **1E-05** | **1E-05** | **1E-05** | **1E-05** | **1E-05** | **1E-05** | **1E-05** | **1E-05** | **1E-05** | **1E-05** | **1E-05** | **1E-05** | **1E-05** | **1E-05** | **1E-05** | **1E-05** | **1E-05** | 0.991 | 0 | 0.002 | 0.065 | 0.067 | 0.073 | 0.068 | 0.140 | 0.112 | 0.107 | 0.117 | 0.126 | 0.078 | 0.149 | 0.122 | 0.133 |
| SXMXB | **1E-05** | **1E-05** | **1E-05** | **1E-05** | **1E-05** | **1E-05** | **1E-05** | **1E-05** | **1E-05** | **1E-05** | **1E-05** | **1E-05** | **1E-05** | **1E-05** | **1E-05** | **1E-05** | **1E-05** | **1E-05** | **1E-05** | **1E-05** | **0.009** | 0.153 | 0 | 0.089 | 0.079 | 0.096 | 0.102 | 0.186 | 0.145 | 0.126 | 0.146 | 0.154 | 0.106 | 0.172 | 0.150 | 0.162 |
| SXFX | **1E-05** | **1E-05** | **1E-05** | **1E-05** | **1E-05** | **1E-05** | **1E-05** | 0.063 | **1E-05** | **1E-05** | **1E-05** | **1E-05** | **1E-05** | **1E-05** | **1E-05** | **1E-05** | **1E-05** | **1E-05** | **1E-05** | **1E-05** | **1E-05** | **1E-05** | **1E-05** | 0 | 0 | 0 | 0.013 | 0.114 | 0.120 | 0.045 | 0.049 | 0.050 | 0.016 | 0.163 | 0.144 | 0.149 |
| SXYS | **1E-05** | **1E-05** | **1E-05** | **1E-05** | **1E-05** | **1E-05** | **1E-05** | 0.108 | **1E-05** | **1E-05** | **1E-05** | 0.063 | **1E-05** | **1E-05** | **1E-05** | **1E-05** | **1E-05** | **1E-05** | **1E-05** | **1E-05** | **1E-05** | **1E-05** | **1E-05** | 0.595 | 0 | 0 | 0.028 | 0.115 | 0.117 | 0.045 | 0.059 | 0.066 | 0.032 | 0.139 | 0.122 | 0.137 |
| SXLFA | **1E-05** | **1E-05** | **1E-05** | **1E-05** | **1E-05** | **1E-05** | **1E-05** | 0.135 | **1E-05** | **1E-05** | **1E-05** | **1E-05** | **1E-05** | **1E-05** | **1E-05** | **1E-05** | **1E-05** | **1E-05** | **1E-05** | **1E-05** | **1E-05** | **1E-05** | **1E-05** | 0.36 | 0.802 | 0 | 0.024 | 0.103 | 0.122 | 0.041 | 0.050 | 0.057 | 0.033 | 0.145 | 0.136 | 0.143 |
| SXLFB | **1E-05** | **1E-05** | **1E-05** | 0.027 | **1E-05** | **1E-05** | 0.117 | 0.18 | **0.009** | 0.018 | 0.568 | 0.117 | 0.126 | 0.144 | 0.018 | 0.018 | 0.018 | 0.198 | **0.009** | 0.126 | **1E-05** | **1E-05** | **1E-05** | 0.054 | **0.009** | 0.027 | 0 | 0.089 | 0.077 | 0.022 | 0.023 | 0.032 | 0.010 | 0.132 | 0.103 | 0.111 |
| BJJF | **1E-05** | **1E-05** | **1E-05** | **1E-05** | **1E-05** | **1E-05** | **1E-05** | **1E-05** | **1E-05** | **1E-05** | **1E-05** | **1E-05** | **1E-05** | **1E-05** | **1E-05** | **1E-05** | **1E-05** | **1E-05** | **1E-05** | **1E-05** | **1E-05** | **1E-05** | **1E-05** | **1E-05** | **1E-05** | **1E-05** | **1E-05** | 0 | 0.046 | 0.095 | 0.093 | 0.108 | 0.074 | 0.208 | 0.206 | 0.194 |
| HBBK | **1E-05** | **1E-05** | **1E-05** | **1E-05** | **1E-05** | **1E-05** | **1E-05** | **1E-05** | **1E-05** | **1E-05** | **1E-05** | **1E-05** | **1E-05** | **1E-05** | **1E-05** | **1E-05** | **1E-05** | **1E-05** | **1E-05** | **1E-05** | **1E-05** | **1E-05** | **1E-05** | **1E-05** | **1E-05** | **1E-05** | **1E-05** | **1E-05** | 0 | 0.112 | 0.114 | 0.131 | 0.075 | 0.195 | 0.171 | 0.167 |
| XXFH | **1E-05** | **1E-05** | **1E-05** | **1E-05** | **1E-05** | **1E-05** | **0.009** | **1E-05** | **1E-05** | **1E-05** | 0.018 | **1E-05** | **1E-05** | **1E-05** | **1E-05** | **1E-05** | **1E-05** | **1E-05** | **1E-05** | **1E-05** | **1E-05** | **1E-05** | **1E-05** | **1E-05** | **1E-05** | **1E-05** | 0.018 | **1E-05** | **1E-05** | 0 | 3E-04 | 0.000 | 0.007 | 0.161 | 0.149 | 0.148 |
| HNSY | **1E-05** | **1E-05** | **1E-05** | **1E-05** | **1E-05** | **1E-05** | **1E-05** | **1E-05** | **1E-05** | **1E-05** | **1E-05** | **1E-05** | **1E-05** | **1E-05** | **1E-05** | **1E-05** | **1E-05** | **1E-05** | **1E-05** | **1E-05** | **1E-05** | **1E-05** | **1E-05** | **1E-05** | **1E-05** | **1E-05** | 0.027 | **1E-05** | **1E-05** | 0.423 | 0 | 0.010 | 0.011 | 0.182 | 0.161 | 0.154 |
| HNLY | **1E-05** | **1E-05** | **1E-05** | **1E-05** | **1E-05** | **1E-05** | **1E-05** | **1E-05** | **1E-05** | **1E-05** | **1E-05** | **1E-05** | **1E-05** | **1E-05** | **1E-05** | **1E-05** | **1E-05** | **1E-05** | **1E-05** | **1E-05** | **1E-05** | **1E-05** | **1E-05** | **1E-05** | **1E-05** | **1E-05** | **0.009** | **1E-05** | **1E-05** | 0.523 | 0.955 | 0 | 0.003 | 0.198 | 0.174 | 0.167 |
| SXSN | **1E-05** | **1E-05** | **1E-05** | **1E-05** | **1E-05** | **1E-05** | **1E-05** | **1E-05** | **1E-05** | **1E-05** | **1E-05** | 0.018 | **1E-05** | **1E-05** | **1E-05** | **1E-05** | **1E-05** | **1E-05** | **1E-05** | **1E-05** | **1E-05** | **1E-05** | **1E-05** | **1E-05** | **1E-05** | **1E-05** | 0.171 | **1E-05** | **1E-05** | 0.153 | 0.072 | 0.315 | 0 | 0.165 | 0.143 | 0.143 |
| SXSLA | **1E-05** | **1E-05** | **1E-05** | **1E-05** | **1E-05** | **1E-05** | **1E-05** | **1E-05** | **1E-05** | **1E-05** | **1E-05** | **1E-05** | **1E-05** | **1E-05** | **1E-05** | **1E-05** | **1E-05** | **1E-05** | **1E-05** | **1E-05** | **1E-05** | **1E-05** | **1E-05** | **1E-05** | **1E-05** | **1E-05** | **1E-05** | **1E-05** | **1E-05** | **1E-05** | **1E-05** | **1E-05** | **1E-05** | 0 | 0.084 | 0.053 |
| SXSLB | **1E-05** | **1E-05** | **1E-05** | **1E-05** | **1E-05** | **1E-05** | **1E-05** | **1E-05** | **1E-05** | **1E-05** | **1E-05** | **1E-05** | **1E-05** | **1E-05** | **1E-05** | **1E-05** | **1E-05** | **1E-05** | **1E-05** | **1E-05** | **1E-05** | **1E-05** | **1E-05** | **1E-05** | **1E-05** | **1E-05** | **1E-05** | **1E-05** | **1E-05** | **1E-05** | **1E-05** | **1E-05** | **1E-05** | **1E-05** | 0 | 0.008 |
| SDHZA | **1E-05** | **1E-05** | **1E-05** | **1E-05** | **1E-05** | **1E-05** | **1E-05** | **1E-05** | **1E-05** | **1E-05** | **1E-05** | **1E-05** | **1E-05** | **1E-05** | **1E-05** | **1E-05** | **1E-05** | **1E-05** | **1E-05** | **1E-05** | **1E-05** | **1E-05** | **1E-05** | **1E-05** | **1E-05** | **1E-05** | **1E-05** | **1E-05** | **1E-05** | **1E-05** | **1E-05** | **1E-05** | **1E-05** | **1E-05** | 0.081 | 0 |
| **Note:** Significance Level=0.001. *F_ST_* values are above the diagonal and associated *P* values below. All *P*-values in bold were judged significant after sequential bonferroni correction. | | | | | | | | | | | | | | | | | | | | | | | | | | | | | | | | | | | | |
